# Regulated degradation of HMG CoA reductase requires conformational changes in sterol-sensing domain

**DOI:** 10.1038/s41467-022-32025-5

**Published:** 2022-07-25

**Authors:** Hongwen Chen, Xiaofeng Qi, Rebecca A. Faulkner, Marc M. Schumacher, Linda M. Donnelly, Russell A. DeBose-Boyd, Xiaochun Li

**Affiliations:** 1 Department of Molecular Genetics, University of Texas Southwestern Medical Center, Dallas, TX USA; 2 Department of Biophysics, University of Texas Southwestern Medical Center, Dallas, TX USA

**Keywords:** Multienzyme complexes, Sterols, Cryoelectron microscopy

## Abstract

3-Hydroxy-3-methylglutaryl coenzyme A reductase (HMGCR) is the rate-limiting enzyme in cholesterol synthesis and target of cholesterol-lowering statin drugs. Accumulation of sterols in endoplasmic reticulum (ER) membranes accelerates degradation of HMGCR, slowing the synthesis of cholesterol. Degradation of HMGCR is inhibited by its binding to UBIAD1 (UbiA prenyltransferase domain-containing protein-1). This inhibition contributes to statin-induced accumulation of HMGCR, which limits their cholesterol-lowering effects. Here, we report cryo-electron microscopy structures of the HMGCR-UBIAD1 complex, which is maintained by interactions between transmembrane helix (TM) 7 of HMGCR and TMs 2–4 of UBIAD1. Disrupting this interface by mutagenesis prevents complex formation, enhancing HMGCR degradation. TMs 2–6 of HMGCR contain a 170-amino acid sterol sensing domain (SSD), which exists in two conformations—one of which is essential for degradation. Thus, our data supports a model that rearrangement of the TMs in the SSD permits recruitment of proteins that initate HMGCR degradation, a key reaction in the regulatory system that governs cholesterol synthesis.

## Abstract (web summary)

HMG-CoA reductase (HMGCR) is regulated by UBIAD1 and Insigs and initializes cholesterol synthesis. Here authors show that the sterol sensing domain of HMGCR undergoes conformational changes to regulate its degradation via binding its protein modulators.

## Introduction

3-Hydroxy-3-methylglutaryl coenzyme A reductase (HMGCR) is a polytopic, endoplasmic reticulum (ER)-localized glycoprotein that catalyzes a rate-limiting step in synthesis of cholesterol and essential nonsterol isoprenoids such as farnesyl pyrophosphate and geranylgeranyl pyrophosphate (GGpp)^1^. HMGCR is tightly controlled by a complex feedback regulatory system that allows cells to constantly synthesize nonsterol isoprenoids while avoiding toxic overproduction of cholesterol and other sterols^2–5^. Part of this feedback control involves accelerated ERAD (ER-associated degradation) of HMGCR^6,7^. This ERAD is initiated by the accumulation of sterols in ER membranes, which triggers binding of HMGCR to ER membrane proteins called Insigs^8,9^. Insig binding is mediated by the N-terminal membrane domain of HMGCR, which is both necessary and sufficient for ERAD and contains eight transmembrane helices (TMs) that precede a large cytosolic catalytic domain^10,11^. TMs 2–6 of HMGCR comprise what is known as the sterol-sensing domain (SSD); mutation of a tetrapeptide sequence (Y_75_IYF) in the SSD of HMGCR abolishes its binding to Insigs, preventing ubiquitination and ERAD^9^. Insig-associated ubiquitin ligases mediate ubiquitination of lysines-89 and −248 (K89 and K248), which are exposed to the cytosol and lie adjacent to TMs 3 and 7 of HMGCR, respectively^9,12–14^. Sterol-induced ubiquitination marks HMGCR for extraction across ER membranes, after which it becomes dislocated into the cytosol for proteasomal degradation^15^. The combination of K89R and K248R mutations prevent sterol-induced ubiquitination and ERAD of HMGCR in both cultured cells and tissues of knock-in mice^9,16^.

**Fig. 1:**
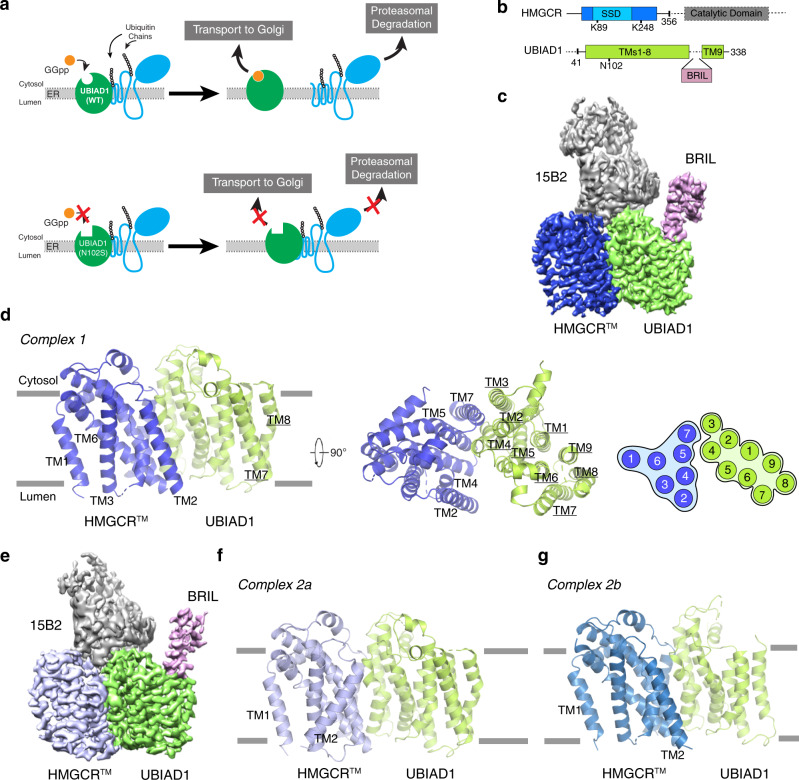
Cryo-EM structure of the HMGCR^TM^-UBIAD1^N102S^ complex. **a** Overview of UBIAD1-mediated regulation of HMGCR ERAD. **b** Schematic representation of HMGCR and UBIAD1 variants used for structural determination. The position of BRIL insertion in UBIAD1 is indicated. **c** Cryo-EM map of HMGCR^TM^-UBIAD1^N102S^ complex 1. **d** Overall structure of HMGCR^TM^-UBIAD1^N102S^ complex 1 viewed from the side of the membrane (left) and cytosol (right). The TMs of UBIAD1 are denoted by underlining. The cartoon denotes a slice of the TMs of HMGCR^TM^ and UBIAD1^N102S^ with helices indicated by numbers. **e** Cryo-EM map of HMGCR^TM^-UBIAD1^N102S^ complex 2a. **f**, **g** Overall structure of HMGCR^TM^-UBIAD1^N102S^ complex 2a **f** and complex 2b **g** viewed from the membrane side.

Whereas Insigs accelerate the ERAD of HMGCR, another protein called UBIAD1 (UbiA prenyltransferase domain-containing protein-1) binds to and stabilizes the enzyme^17,18^. UBIAD1 was discovered as the enzyme that transfers the geranylgeranyl group from GGpp to menadione producing the vitamin K_2_ subtype menaquinone-4 (MK-4)^19,20^. We subsequently identified UBIAD1 as a GGpp sensor that binds to HMGCR and inhibits its ERAD when ER membranes are depleted of GGpp^17^. When GGpp accumulates within ER membranes, the isoprene binds to UBIAD1, causing it to dissociate from HMGCR and translocate to the medial*-trans* cisternae of the Golgi^17,21^. Importantly, GGpp-induced translocation of UBIAD1 from the ER-to-Golgi occurs in HMGCR-deficient cells^22^. Dissociation from UBIAD1 allows for the maximal ERAD of HMGCR (Fig. 1a). The physiologic significance of the UBIAD1-HMGCR interaction is confirmed by the observation that missense mutations in UBIAD1 cause Schnyder corneal dystrophy (SCD), an autosomal-dominant eye disease characterized by corneal opacification owing to the over-accumulation of cholesterol^23,24^. SCD-associated variants of UBIAD1 are sequestered in the ER and resist GGpp-induced dissociation from HMGCR (Fig. 1a). As a result, SCD-associated UBIAD1 inhibits ERAD of HMGCR, which leads to enhanced synthesis and accumulation of cholesterol in both cultured cells and tissues of mice^17,21,22,25^.

Competitive inhibitors of HMGCR called statins are prescribed to lower circulating levels of low density lipoprotein (LDL)-cholesterol and reduce the incidence of atherosclerotic cardiovascular disease (ACVD)^26,27^. However, the efficacy of statins is reduced because they disrupt feedback control of HMGCR owing to depletion of sterol and nonsterol isoprenoids (including GGpp). This depletion leads to the accumulation of HMGCR in the liver that overcomes inhibitory effects of statins, allowing continued synthesis of cholesterol that limits lowering of plasma cholesterol^28–32^. Our previous studies indicated that inhibition of ERAD substantially contributes to statin-induced accumulation of HMGCR, which correlates with ER sequestration of UBIAD1^16,25^. However, the molecular basis through which sterols and GGpp accelerate ERAD of HMGCR and how UBIAD1 blocks the reaction is unknown. Here, we determined cryogenic electron microscopy (cryo-EM) structures of HMGCR bound to SCD-associated UBIAD1 (N102S). Structural and functional analysis provide key insights into mechanisms for UBIAD1-mediated protection of HMGCR from ERAD. These findings have important implications for development of agents that enhance statin efficacy and further reduce ACVD. Moreover, our studies reveal that the HMGCR SSD adopts a specific conformation required for sterol-accelerated ERAD, establishing the molecular basis through which the region mediates regulation of cholesterol synthesis.

## Results

### Assembly of a complex between HMGCR and UBIAD1

We focused on the stabilizing interaction between the membrane domain of hamster HMGCR and UBIAD1. The hamster proteins share over 95% overall identity with their human counterparts (Supplementary Fig. 1). Expression plasmids were prepared that encode the FLAG-tagged membrane domain of HMGCR harboring arginine substitutions for K89 and K248 (designated HMGCR^TM^) and Strep-tagged UBIAD1 from which we deleted a flexible N-terminal region (amino acids 1–40). We included the SCD-associated N102S mutation, which blunts enzymatic activity^20,33^; this protein is designated UBIAD1^N102S^ (Fig. 1b). Notably, UBIAD1 containing the N-terminal deletion continued to localize to the Golgi of GGpp-replete cells (Supplementary Fig. 2), indicating the protein was normally folded. HMGCR^TM^ and UBIAD1^N102S^ were co-expressed in HEK-293 GnTI^-^ cells and purified by anti-FLAG chromatography. Gel filtration shows that the HMGCR^TM^-UBIAD1^N102S^ complex migrated as a single peak (Supplementary Fig. 3a); the presence of both proteins in the peak fraction was confirmed by immunoblot and mass spectrometry. Unfortunately, cryo-EM images of the HMGCR^TM^-UBIAD1^N102S^ complex displayed limited features and we failed to reconstitute a 3D model.

Hydropathy plots predict that UBIAD1 is comprised of 9 TMs. To provide a fiducial marker for particle image alignment in cryo-EM structure determination, we inserted the soluble, thermostabilized apocytochrome b562RIL (BRIL)^34^ in a cytosolic loop predicted to localize between TMs 8 and 9 of UBIAD1 (designated UBIAD1-BRIL^N102S^) (Fig. 1b). The yield and biochemical stability of the HMGCR^TM^-UBIAD1-BRIL^N102S^ was considerably enhanced compared to the HMGCR^TM^-UBIAD1^N102S^ complex. HMGCR^TM^-UBIAD1-BRIL^N102S^ was assembled into complex with anti-BRIL Fab (Fab^BRIL^)^34^ and an anti-Fab nanobody (Nb)^35^ in amphipols (Supplementary Fig. 3c, d), of which the structure was determined by cryo-EM at a resolution of 3.6-Å (Supplementary Fig. 4). Densities of all 9 TMs of UBIAD1 and TMs 1–7 of HMGCR were resolved. However, the cryo-EM map of HMGCR part does not provide sufficient resolution to determine the atomic structure (Supplementary Fig. 4c). To address this problem, we generated and screened ∼1000 hybridoma clones for conformation-specific antibodies using HMGCR^TM^-UBIAD1^N102S^ complex as antigen. We identified one monoclonal antibody designated IgG-15B2 that bound native HMGCR^TM^-UBIAD1^N102S^, and found that BRIL insertion did not interfere the epitope recognition. Fab^15B2^, a Fab fragment derived from IgG-15B2, was co-purified with HMGCR^TM^-UBIAD1-BRIL^N102S^ upon gel filtration (Supplementary Fig. 3b, e).

The structure of the HMGCR^TM^-UBIAD1-BRIL^N102S^-Fab^15B2^ complex was determined by cryo-EM at a resolution of 3.3-Å (Fig. 1c, g, Supplementary Table 1). HMGCR^TM^-UBIAD1-BRIL^N102S^-Fab^15B2^ complex existed as either a monomeric or dimeric heterotrimer. We observed clear densities for all 9 TMs of UBIAD1 and TMs 1-7 of HMGCR in the monomeric HMGCR^TM^-UBIAD1-BRIL^N102S^-Fab^15B2^ heterotrimer (designated UBIAD1-HMGCR complex 1) (Fig.  1c, d, Supplementary Figs. 5, 6). Interestingly, within the dimeric structure of the HMGCR^TM^-UBIAD1-BRIL^N102S^-Fab^15B2^ heterotrimer, each HMGCR^TM^ in the complex existed in a different conformational state. The structure of one state, designated UBIAD1-HMGCR complex 2a, was determined at a resolution similar to that of complex 1 (Fig. 1f, Supplementary Figs. 5, 7). The structure of the other state, designated UBIAD1-HMGCR complex 2b, was resolved at a lower resolution. We observed clear cryo-EM map for TM1-7 of HMGCR in complex 2b (Supplementary Fig. 7a); however, maps corresponding to TMs 7-9 of UBIAD1, BRIL, and Fab^15B2^ failed to be observed. The cryo-EM map shows that the HMGCR^TM^-UBIAD1-BRIL^N102S^-Fab^15B2^ complex 2b was rotated 180° in detergent compared to complex 2a (Supplementary Fig. 7a). This rotation is likely an artifact of detergent solubilization and unlikely to be physiological.

### Overall Structure of the UBIAD1-HMGCR complex

Cryo-EM maps revealed that the Fab^15B2^ epitope encompasses the cytosolic interface of the HMGCR^TM^-UBIAD1-BRIL^N102S^ complex and includes regions of both proteins (Fig. 1c, e, Supplementary Fig. 8). The possibility exists that Fab^15B2^ modulates complex formation by altering the structure of HMGCR^TM^ and/or UBIAD-BRIL^N102S^. Structural analysis of HMGCR^TM^-UBIAD1-BRIL^N102S^-Fab^15B2^ complex indicates that Fab^BRIL^ and Nb disrupted the dimer interface (Supplementary Fig. 9a, b); thus, the HMGCR^TM^-UBIAD1-BRIL^N102S^-Fab^BRIL^-Nb complex was only observed in the monomeric state. Although the overall resolution of HMGCR^TM^-UBIAD1-BRIL^N102S^-Fab^BRIL^-Nb complex is lower than that of the HMGCR^TM^-UBIAD1-BRIL^N102S^-Fab^15B2^ complex, structural analysis revealed the UBIAD1-HMGCR interface was identical in both complexes (Supplementary Figs. 4 and 9c).

**Fig. 2:**
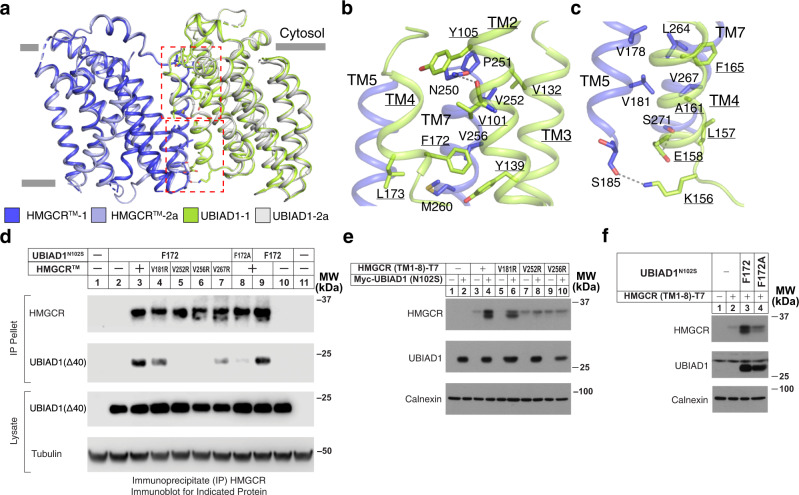
Analysis of the HMGCR^TM^-UBIAD1^N102S^ complex interface. **a** Structural comparison of HMGCR^TM^-UBIAD1^N102S^ complex 1 and complex 2a. HMGCR-TM2 adopts distinct conformations in the two complexes. TMs that comprise the interface of the HMGCR-UBIAD1 are indicated dashed red boxes. Detailed interactions in the upper and lower dashed boxes are shown individually in **b**, **c**, respectively. Residues that contribute to complex formation are labeled; hydrophilic interactions are indicated by dashed lines. **d** Detergent lysates of HEK-293 GnTI^-^ cells transfected with expression plasmids encoding FLAG-tagged HMGCR^TM^ and Strep-tagged UBIAD1^N102S^ were precipitated with anti-FLAG-M2 agarose beads. Aliquots of resulting precipitates and lysates were subjected to SDS-PAGE, followed by immunoblot analysis. Experimental details are provided in “Methods”. **e**, **f** SV-589 (ΔUBIAD1) cells transfected with expression plasmids encoding indicated variant of HMGCR (TM1-8)-T7 and Myc-UBIAD1 (N102S) were lysed and subjected to subcellular fractionation. Aliquots of resulting membrane fractions were then subjected to SDS-PAGE and immunoblot analysis with anti-FLAG or anti-T7 (for HMGCR) and anti-Strep or anti-Myc (IgG-9E10, for UBIAD1). Additional experimental details can be found in “Methods”. Source data are provided as a Source Data file.

The conformation of UBIAD1 is identical in Complexes 1 and 2a (Fig. 2a). The interface between UBIAD1 and HMGCR occupies an area of ~1000 Å^2^; the complex is maintained by several interactions between TMs 2 and 4 of UBIAD1 and TMs 5 and 7 of HMGCR (Figs. 1d and 2a). The main chain of V101, which localizes to TM2 of UBIAD1, makes hydrophilic contact with N250 in TM7 of HMGCR (Fig. 2b). TM4 of UBIAD1 makes additional hydrophobic contacts with HMGCR and two hydrophilic interactions are observed between E158 of UBIAD1-TM4 and S271 of HMGCR-TM7 and between K156 of UBIAD1-TM4 and the main chain in S185 of HMGCR-TM5 (Fig. 2c). A hydrophobic interface is formed by interaction of residues in TM5 and TM7 of HMGCR with residues in TM2, TM3, and TM4 of UBIAD1 (Fig.  2b, c).

We next compared the association of UBIAD1^N102S^ with HMGCR^TM^ and variants of the protein harboring mutations in the HMGCR-UBIAD1 interface predicted to disrupt complex formation (V181R, V252R, V256R, and V267R in HMGCR) (Fig. 2b, c). Because the HMGCR-UBIAD1 complex cannot be assembled in vitro, we used co-immunoprecipitation of the proteins expressed in vivo to measure their association. HEK-293 GnTI^-^ cells transfected with expression plasmids encoding Strep-tagged UBIAD1^N102S^ and FLAG-tagged HMGCR^TM^ or its variants were lysed and precipitated with anti-FLAG-coupled agarose beads. Immunoblot analysis of precipitated material revealed that as expected, UBIAD1^N102S^ co-precipitated with HMGCR^TM^ (Fig. 2d, lane 3). In contrast, UBIAD1^N102S^ failed to be co-precipitated with HMGCR^TM^ (V252R) and HMGCR^TM^ (V256R) (lanes 5 and 6); HMGCR^TM^ (V181R) and HMGCR^TM^ (V267R) precipitated UBIAD1^N102S^ albeit at slightly reduced levels compared to HMGCR^TM^ (lanes 4 and 7). We also generated a variant of UBIAD1^N102S^ containing a point mutation (F172A) at the HMGCR-UBIAD1 complex interface (Fig. 2b, c). The results show that co-precipitation of UBIAD1^N102S^ (F172A) with HMGCR^TM^ was reduced compared to UBIAD1^N102S^ (compare lane 8 with lanes 3 and 9).

When transfected into UBIAD1-deficient cells, the amount of the T7-tagged membrane domain of wild type HMGCR (HMGCR (TM1-8)-T7) was low (Fig. 2e, lane 3). This is consistent with our previous observation that in both cultured cells and whole animals, ERAD of HMGCR was accelerated in the absence of UBIAD1^21,36^. Co-expression of full-length, Myc-tagged UBIAD1^N102S^ markedly stabilized HMGCR (TM1-8)-T7 as expected (Fig. 2e, lane 4). HMGCR^V181R^ (TM1-8)-T7 was similarly stabilized in the presence of Myc-UBIAD1^N102S^ (Fig. 2e, lanes 5 and 6). However, HMGCR^V252R^ (TM1-8)-T7 and HMGCR^V256R^ (TM1-8)-T7 failed to become stabilized in the presence of Myc-UBIAD1^N102S^ (Fig. 2e, lanes 7–10). Our structural observations were further supported by the finding that UBIAD1 (N102S/F172A) failed to stabilize HMGCR (TM1-8)-T7 to the extent observed with Myc-UBIAD1 (N102S) (Fig. 2f, lanes 3 and 4).

### Structural analysis of UBIAD1^N102S^

**Fig. 3:**
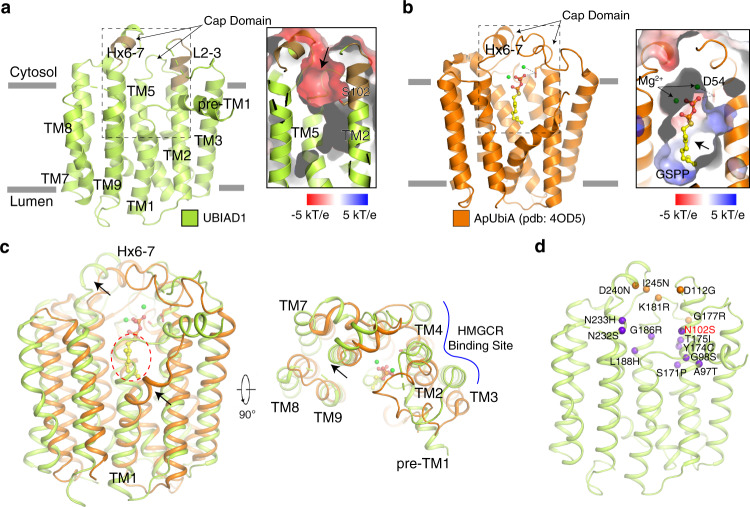
Structural analysis of UBIAD1^N102S^. **a** Overall structure of UBIAD1 viewed from the side of the membrane. N_102_TYYDFSKG and D_236_MESD motifs are colored in brown and cytosolic loops that form the Cap Domain are indicated by arrows. The electrostatic surface representation of the catalytic cavity is shown on the right. The entrance of the catalytic cavity is indicated by an arrow. The N102S mutation is shown in sticks. **b** Overall structure of the UbiA homolog from *Aeropyrum pernix* (ApUbiA) viewed from the side of the membrane. The electrostatic surface representation of the catalytic cavity is shown on the right. The entrance of the catalytic cavity is indicated by an arrow. The putative substrate GSPP is shown in sticks and the Mg^2+^ ions are shown in green balls. **c** Structural comparison of UBIAD1 and ApUbiA. The movements of TM1 and Hx6-7 are indicated by arrows. The potential clash between UBIAD1-TM1 and GSPP is indicated by a dashed oval. The HMGCR binding site is indicated by a blue line. **d** Distribution of SCD-associated mutation in UBIAD1. The mutations that locate in the cap domain are colored in orange and the mutations that locate in the catalytic core are colored in purple.

Despite limited sequence similarities, the overall structure of hamster UBIAD1^N102S^ resembles the previously reported structures of two archaeal UbiA prenyltransferases^37,38^ with a root-mean-square-deviation (RMSD) of 3.1 Å (Fig. 3a–c). Loops that separate the TM helices are relatively short except for the loop between TMs 2 and 3 (L2-3; amino acids 108–129) and the helix between TMs 6 and 7 (Hx6-7; amino acids 235–243). UbiA prenyltransferases contain two aspartate-rich motifs (NDXXDXXXD and DXXD) that are essential for enzymatic activity^39^. These motifs, which correspond to N_102_TYYDFSKG and D_236_MESD in UBIAD1, are located at the C-terminal ends of TMs 2 and 6, respectively (Fig. 3a). Hx6-7, L2-3, and the loop between TMs 4 and 5 (L4-5) form a cap domain that lies over a central, negatively charged cavity generated by TMs 1, 2, 4, 5, and 6 (Fig. 3a). The structural analysis of the archaeal UbiA prenyltransferases suggests a model in which the cap domain adopts an open conformation in the absence of the isoprenyl substrate^37,38^. Binding of the substrate induces conformational changes in L2-3 that causes the cap domain to adopt a closed conformation that seals the enzyme’s active site (Fig. 3b). The structure of UBIAD1^N102S^ reveals that the cap domain adopts an open conformation, which is consistent with previous findings that the N102S mutation reduces the affinity of UBIAD1 for GGpp^20^. Figure 3d shows the location of residues in UBIAD1 that are mutated in SCD. SCD-associated mutations cluster in L2-3 and Hx6-7 or line the central cavity that harbors the enzyme’s active site. These residues, many of which are conserved in UbiA prenyltransferases, are likely involved in binding of GGpp or catalysis. Indeed, our group and others have demonstrated that introduction of SCD-associated mutations in UBIAD1 reduces enzymatic activity^20,33^.

In comparing the structures of archaeal UbiA prenyltransferases (bound to substrate) and UBIAD1^N102S^, we noticed a significant difference. The central cavity in the archaeal enzymes has a lateral opening delineated by kinked TM1 and TM9^38^ (Fig. 3b, c). This lateral opening may allow these enzymes to accommodate longer isoprenyl substrates and/or release reaction products. Interestingly, TM1 of UBIAD1^N102S^ forms an intact α-helix that blocks the lateral opening (Fig. 3a, c). It is tempting to speculate that binding to GGpp or MK-4 triggers conformational changes in TM1 that allows release of the product into the membrane bilayer.

### Structural analysis of HMGCR^TM^

**Fig. 4:**
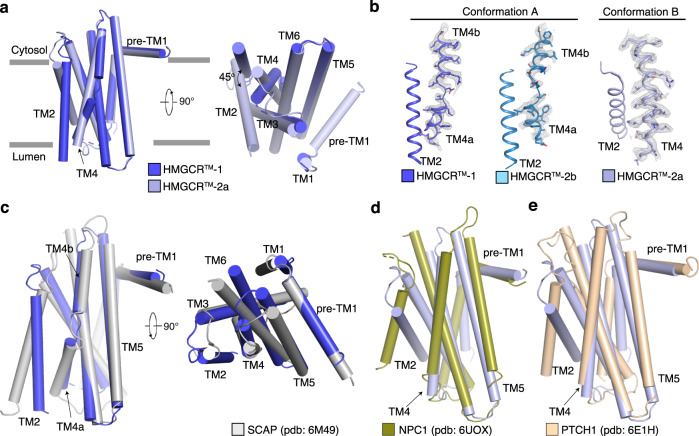
Structural analysis of HMGCR^TM^. **a** Structural comparison of HMGCR^TM^-1 and HMGCR^TM^-2a viewed from the side of the membrane (left) and lumen (right). **b** Structures of HMGCR-TM2 and TM4 in HMGCR^TM^-UBIAD1^N102S^ complexes 1, 2a and 2b. The cryo-EM maps of TM4 are shown as gray mesh. **c**–**e** Structural comparison of HMGCR-TMs 1–6 (Conformation A) to SCAP-TMs 1–6 **c**, HMGCR-TMs 1–6 (Conformation B) to NPC1-TMs 2–7 **d**, and HMGCR-TMs 1–6 (Conformation B) to PTCH1-TMs 1–6 **e**. HMGCR-TM4 is indicated by arrows.

The overall structure of HMGCR^TM^ revealed that TMs1–7 are integrated into membranes. The loops between TM1 and TM2 (25 amino acids in length) as well as TM8 were not visualized in the structure, which indicates considerable flexibility within the regions (Fig. 1d). Further analysis of the resolved structure revealed that HMGCR^TM^ adopts two distinct conformations designated Conformation A and Conformation B (Fig. 4a, b). In UBIAD1-HMGCR complex 1 and 2b, HMGCR^TM^ assumes Conformation A in which TM2 is perpendicular to the membrane, whereas TM4 is unwound to generate two half helices that we designate TM4a and TM4b (Fig. 4b). HMGCR^TM^ assumes Conformation B in UBIAD1-HMGCR complex 2a. TM2 is tilted 45° in the membrane (Fig. 4a) and TM4 forms an intact α-helix (Fig. 4b). In the dimeric state of complex 2a, Conformation B becomes stabilized through direct interactions between TMs of HMGCR.

SSDs are found in five other proteins—Scap, Niemann-Pick C1 (NPC1), NPC1-Like1 (NPC1L1), Patched, and Dispatched—implicated in the regulation of cholesterol metabolism and signaling (Supplementary Fig. 10). Scap is a cholesterol-regulated escort protein required for activation of membrane-bound transcription factors called sterol regulatory element-binding proteins (SREBPs)^40^. NPC1 and NPC1L1 mediate intracellular transport of LDL-derived or dietary cholesterol^41^. Patched binds to the cholesterol-modified morphogen Hedgehog, while Dispatched mediates release of Hedgehog from cells^42^. Scap and HMGCR are unique among SSD-containing proteins in that sterols cause both proteins to bind Insigs. However, Insig binding does not lead to accelerated ERAD of Scap. Instead, the reaction traps Scap in the ER, preventing its transport to the Golgi for proteolytic activation of bound SREBPs^43^.

The structures of NPC1, NPC1L1, Patched, Dispatched, and Scap in complex with Insig-2, have been determined^44–53^. TMs 2–6 constitute the SSD in HMGCR, Scap, and Patched, whereas TMs 3–7 constitutes the NPC1-SSD and NPC1L1-SSD. The TMs of NPC1, NPC1L1, Patched, and Dispatched contain at least 12 transmembrane helices including a pseudo-SSD that associates with the SSD to restrain its conformation (Supplementary Fig. 11). We compared the structures of HMGCR (TMs 1–6), Scap (TMs 1–6), NPC1 (TMs 2–7) and Patched (TMs 1–6). These comparisons revealed the structure of HMGCR (TMs 1–6) in Conformation A is similar to that of Insig-bound Scap (Fig. 4c). TM2 in both SSDs is vertical in the membrane and remarkably, TM4 is broken at similar positions (Fig. 4c, Supplementary Fig. 10). The tilted conformation of HMGCR-TM2 is not observed in corresponding TMs of NPC1 (TM3) and Patched (TM2). NPC1-TM5 and Patched-TM4 correspond to HMGCR-TM4 and resemble its configuration when HMGCR^TM^ assumes Conformation B (Fig. 4d, e).

### Dynamic Reorganization of TMs in the HMGCR SSD

**Fig. 5:**
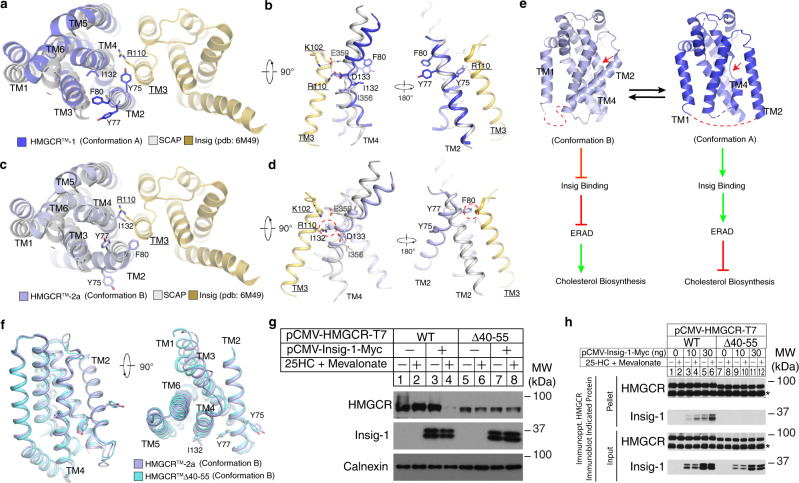
The structural comparison of HMGCR to Insig-bound Scap reveals dynamic features of the SSD. **a** HMGCR-TMs 1-6 (Conformation A) docked to the structure of the Scap-Insig complex. The residue Y75 and Y77 of YIYF motif is shown in sticks. **b** HMGCR-TMs 1–6 (Conformation A) docked to the structure of SCAP-Insig complex. The TMs of Insig are indicated by underlining. Interactions between amino acids in Scap and Insig are indicated by dashed lines. **c** HMGCR-TMs 1-6 (Conformation B) docked to the structure of the Scap-Insig complex. **d** HMGCR-TMs 1-6 (Conformation B) docked to the structure of SCAP-Insig complex. The conformation of TM2 when HMGCR adopts Conformation B interferes with the putative interaction between HMGCR and Insig. The clashes between Insig R110 and HMGCR-I132; Insig-TM3 and HMGCR-F80 are indicated by dashed circles. **e** L1–2 regulates the conformational transition of HMGCR-SSD. L1-2 is indicated by the red dashed lines. **f** Structural comparison of HMGCR^TM^-2a and HMGCR^TM^ (Δ40–55). **g** HMGCR-deficient Chinese hamster ovary (CHO) cells transfected with expression plasmids encoding indicated variant of HMGCR-T7 and Insig-1-Myc were treated in the absence or presence of 1 µg/ml 25-HC and 10 mM mevalonate and were then harvested and lysed. Aliquots of whole cell lysates were subjected to SDS-PAGE, followed by immunoblot analysis with IgG-A9 (against HMGCR) or anti-Myc (IgG-9E10, against Insig-1). **h** HMGCR-deficient CHO cells transfected with expression plasmids encoding the indicated HMGCR variant and Insig-1 were depleted of sterols for 16 h. The cells were then pretreated with the proteasome inhibitor MG-132 for 1 h, followed by treatment with 1 µg/ml 25-HC for 30 min. Cells were then harvested, lysed, and subjected to immunoprecipitation followed by immunoblot analysis with anti-T7 (against HMGCR) or anti-Myc (IgG-9E10, against Insig-1). Asterisks indicate nonspecific bands. Additional experimental details can be found in “Methods”. Source data are provided as a Source Data file.

Further analysis of the two HMGCR SSD conformations may provide insight into mechanisms for the sterol-sensing reaction. Thus, we superimposed the structure of TMs 1–6 of HMGCR in Conformation A with the previously reported structure of the Scap-Insig-2 complex^50^. This superimposition indicates that the predicted HMGCR-Insig-2 interface is similar to the Scap-Insig interface, which is comprised of TMs 2, 4, and 5 of Scap and TMs 3 and 4 of Insig-2 (Fig. 5a). A previous finding suggested that unwinding of Scap-TM4 exposes negatively charged E359, permitting interaction with R110 (and perhaps K102) of Insig-2 that stabilizes the Scap-Insig-2 complex^50^. Our modeling predicts that D133 of HMGCR assumes a position equivalent to that of E359 in the broken TM4 of Scap and contributes to formation of the HMGCR-Insig-2 complex (Fig. 5b). The YIYF motif of Scap and HMGCR, which is required for their sterol-induced binding to Insigs^50^, is positioned similarly in TM2 of both proteins (Fig. 5b).

Figure 5c shows the superimposed structures of the Scap-Insig-2 complex and HMGCR (TMs 1–6) in Conformation B that results in rotation of TM2 and TM4 approximately 180° (Fig. 5a, c). This rotation causes significant steric hinderance between F80 and Insig-2-TM3; steric clash is also observed between I132 in HMGCR and R110 of Insig-2 (Fig. 5c, d). It is notable that in the absence of Insig-2, Scap-TM2 is not observed in the cryo-EM maps, which indicates considerable flexibility. TM4 is not broken and may adopt a continuous α-helix similar to that of HMGCR-TM4 in Conformation B (Supplementary Fig. 12)^49^.

Comparing the structure of HMGCR to that of Scap and other SSD-containing proteins led us to speculate that when HMGCR adopts Conformation B, the SSD cannot bind to Insigs and resists ERAD. However, adoption of Conformation A promotes binding of HMGCR to Insig for subsequent ubiquitination and ERAD. Analysis of the HMGCR structure led us to postulate that flexibility of the lumenal loop between TM1 and TM2 (L1–2) may affect the conformation of TM2, leading to reorganization of TMs within the SSD that regulates HMGCR ERAD (Fig. 5e). Thus, we screened several variants of HMGCR haboring deletions within L1-2 that reduce flexibity and restrict the conformation of the SSD. We identified one variant, designated HMGCR^TM^ (Δ40–55), harboring a 16-amino acid deletion that exhibits reasonable expression yield and sufficient biochemical behavior. Cryo-EM analysis of the HMGCR^TM^ (Δ40–55)-UBIAD1^N102S^ complex revealed that the L1-2 deletion caused HMGCR to exclusively assume Conformation B in which TM2 is titled in the membrane; TM4 is intact regardless of its monomeric and dimeric state (Fig. 5f, Supplementary Figs. 3f, 13). To validate these structural observations, we examined the sterol-accelerated ERAD of T7-tagged HMGCR harboring the L1-2 deletion (designated HMGCR-T7 (Δ40–55)). HMGCR-T7 (WT) was subjected to Insig-mediated ERAD stimulated by the oxysterol 25-hydroxycholesterol (25-HC) and mevalonate (which provides a source of GGpp) (Fig. 5g, lanes 1–4). In contrast, HMGCR-T7 (Δ40–55) completely resisted 25-HC-induced ERAD (Fig. 5g, lanes 5–8). Co-immunoprecipitation was used to measure sterol-mediated association of HMGCR-T7 (WT) and (Δ40–55) with Insig-1. The results show that 25-HC enhanced the co-precipitation of Insig-1 with HMGCR-T7 (WT) (Fig. 5h, lanes 1-6), but not HMGCR-T7 (Δ40–55) (Fig. 5h, lanes 7-12). Based on these findings, we conclude that flexibility of L1-2 significantly contributes to reorganization of the SSD that permits binding of Insigs.

## Discussion

Previous studies have described an intricate pathway through which distinct lipids—sterols and GGpp—accelerate Insig-mediated ERAD of HMGCR^3^. A key breakthrough in the understanding of HMGCR ERAD came with the discovery that UBIAD1 binds to HMGCR and inhibits its ERAD. In the current studies, we analyze the structure of the HMGCR-UBIAD1 complex, which reveals a hydrophobic interface that is mediated by multiple interactions between the TMs of UBIAD1 and HMGCR (Fig. 1c–g). Mutation of key residues in this interface disrupts formation of the HMGCR-UBIAD1 complex and blunts UBIAD1-mediated stabilization of HMGCR (Fig. 2d–f). Identification of the HMGCR-UBIAD1 interface has important implications for the molecular basis of statin-induced accumulation of HMGCR that was described more than 40 years ago^2^. Studies in genetically-manipulated mice revealed that inhibition of ERAD substantially contributes to statin-accumulation of HMGCR^16^. UBIAD1 is sequestered in the ER of hepatic membranes isolated from statin-fed mice owing to depletion of GGpp^25^ and unequivocal genetic evidence has been obtained that UBIAD1 is an inhibitor of HMGCR ERAD^36^. Taken together with the current study, we predict molecules that disrupt the HMGCR-UBIAD1 interface or mimic GGpp in stimulating ER-to-Golgi translocation of UBIAD1 will enhance ERAD of HMGCR, preventing its accumulation associated with statin therapy.

We captured the SSD of HMGCR in two distinct conformations, indicating TMs in the region undergo dynamic reorganization within ER membranes (Fig. 5e). Importantly, the cytosolically-exposed sites for sterol-induced ubiquitination (K89 and K248) are identical in both conformations, ruling out the possibility that reorganization of the SSD alters access of the sites to ubiquitination machinery. We attenuated the structural rearragnment of TMs in the SSD through protein engineering and discovered that confining HMGCR to Conformation B (Fig. 5f) abolished its Insig-mediated ERAD stimulated by 25-HC (Fig. 5g). Certain oxysterols such as 25-HC are known to bind Insigs and it has been postulated that oxysterol-bound Insig in turn, associates with the SSDs of Scap and HMGCR^40^. Taking our current study into consideration, it is reasonable to speculate that oxysterol-bound Insig only engages the SSD when it assumes Conformation A. It should be also noted that the cholesterol synthesis intermediate 24,25-dihydrolanosterol (DHL) accelerates Insig-mediated ERAD of HMGCR^3^, but does not bind to Scap or Insig. We hypothesize that the SSD of HMGCR directly senses the concentration of DHL embedded in ER membranes, causing the protein to change its conformation to engage Insigs for ubiquitination and ERAD to control the synthesis of cholesterol. Although the structures of other SSD-containing proteins have been determined^44–53^, these studies neither observed the dynamic reorganization of the SSD nor determined whether multiple conformations of the SSD modulated the protein’s activity. Our findings provide structural evidence validated by functional assays that are beginning to disclose the molecular basis through which SSDs control protein function.

Despite the advance in the understanding of the HMGCR ERAD pathway, several questions remain outstanding. For example, molecular mechanisms underlying transition of the HMGCR-SSD between Conformations A and B regulated by DHL and oxysterols remain to be determined. The precise mechanism through which UBIAD1 inhibits HMGCR ERAD at a post-ubiquitination step in the reaction has not been elucidated. Finally, mechanisms whereby GGpp dissociates the HMGCR-UBIAD1 complex and stimulates ER-to-Golgi transport of UBIAD1 is unknown. Attempts to address these important questions utilizing a variety of approaches are currently underway.

## Methods

### Expression plasmids

A cDNA encoding the transmembrane domain (amino acids 1–356) of hamster HMGCR harboring mutations (K89R, K248R) that abolish the enzyme’s sterol-induced ubiquitination was cloned into the pEZT-BM vector^54^ with a N-terminal FLAG tag. The resulting expression plasmid is designated pEZT-BM-FLAG-HMGCR^TM^. The cDNA for hamster UBIAD1 containing a 40-amino acid deletion at N-terminus and the SCD-associated N102S mutation was cloned into pEZT-BM vector preceded by a StrepII tag. BRIL insertion was screened through different replacement for loops between TMs of UBIAD1. The final construct used for cryo-EM structure determination is the one with BRIL insertion located between Phe300 and Pro309. This expression plasmid is designated pEZT-BM-StrepII-UBIAD1-BRIL^N102S^. The following expression plasmids were described in the indicated reference: pCMV-HMGCR (TM1-8)-T7 encoding amino acids 1–346 of hamster HMGCR with 3 copies of the T7 epitope at the C-terminus under transcriptional control of the cytomegalovirus (CMV) promoter^8^; pCMV-Myc-UBIAD1 (N102S) encoding human UBIAD1 containing a Myc tag at the N-terminus under control of the CMV promoter^17^; pCMV-Insig-1-Myc, which encodes human Insig followed by six copies of the Myc epitope under control of the CMV promoter^43^; and pCMV-HMGCR-7, encoding full-length hamster HMGCR followed by three copies of the T7 epitope under control of the CMV promoter^9^. Site-directed mutagenesis of pCMV-HMGCR (TM1-8)-T7, pEZT-BM-FLAG-HMGCR^TM^, and pEZT-BM-StrepII-UBIAD1^N102S^ (without BRIL insertion) was carried out by two-step overlapping PCR. All mutations were verified by sequencing.

### Cloning, expression, and purification of HMGCR^TM^-UBIAD1^N102S^ complex

pEZT-BM-FLAG-HMGCR^TM^ and pEZT-BM-StrepII-UBIAD1-BRIL^N102S^ were introduced into HEK-293S GnTI^−^ cells (ATCC) by baculovirus-mediated transduction. Following incubation for 60 h at 30 °C, cells were harvested and disrupted by sonication in buffer A (20 mM HEPES pH 7.5, 150 mM NaCl) containing 1 mM PMSF, 10 μg/mL leupeptin. After low-speed centrifugation, the resulting supernatant was incubated with 1% (w/v) lauryl maltose neopentyl glycol (LMNG, Anatrace), 0.1% cholesteryl hemisuccinate (CHS, Steraloids) at 4 °C for 1 h. Lystates were clarified by centrifugation at 20,000 × *g*; the supernatant of this spin was loaded onto an anti-FLAG M2 affinity column (Sigma). After washing twice with buffer B (20 mM HEPES pH 7.5, 400 mM NaCl, 0.1% LMNG, 0.01% CHS, 10 μg/ml POPS and 10 μg/ml Soybean Polar Lipid Extract), bound proteins were eluted in buffer C (20 mM HEPES pH 7.5, 150 mM NaCl, 0.1% LMNG, 0.01% CHS, 10 μg/ml POPS, 10 μg/ml Soybean Polar Lipid Extract and 0.1 mg/ml FLAG peptide). The sample was concentrated and further purified by Superose-6 size-exclusion chromatography (GE Healthcare) in buffer D (20 mM HEPES pH 7.5, 150 mM NaCl and 0.06% Digitonin). The peak fractions were collected for complex assembly. The HMGCR^TM^ (Δ40–55)-UBIAD1-BRIL^N102S^ complex was expressed and purified using the identical procedure.

### Expression and purification of anti-BRIL Fab and its nanobody

The anti-BRIL Fab (Fab^BRIL^) was expressed in *E. coli* and purified as described^34^. The DNA sequence encoding the anti-Fab^BRIL^ nanobody (Nb) was derived from PDB: 6WW2^55^ and synthesized from Integrated DNA Technologies and cloned into pET-26b vector with a C-terminal 6×His tag. The nanobody was expressed in *E. coli* BL21-Gold (DE3) cells with 0.5 mM IPTG at 25 °C overnight and purified by Ni-NTA chromatography and size-exclusion chromatography on a Superdex 200 column. The proteins were concentrated and frozen at −80 °C for further use.

### Generation of Fab^15B2^

Monoclonal antibody (designated 15B2) of isotype IgG1 were raised in mice (NZBWF1/J, The Jackson Laboratory) at the Department of Molecular Genetics of the University of Texas Southwestern Medical Center. The antigen immunize mice was the HMGCR^TM^-UBIAD1^N102S^ complex that was reconstituted into Amphipol A8-35. Hybridomas were created by fusion of splenic B lymphocytes from hyperimmune mice to SP2-mIL6 mouse myeloma cells (ATCC, CRL-2016). We used the combined techniques of ELISA, immunoblot analysis, and immunoprecipitation to identify antibodies that preferentially bound to HMGCR^TMs^-UBIAD1^N102S^ complex in its native, but not SDS-denatured state. These efforts yielded a mouse monoclonal antibody designated IgG-15B2. To clone IgG-15B2, total RNA was isolated from the hybridoma by RNA extraction kit (Qiagen) following the manufacturer’s protocol. Total RNA was subjected to reverse transcription reactions using Superscript III reverse transcription kit (Invitrogen) and the resultant cDNA was used as a template in PCR reactions with degenerate primers to amplify the variable regions. Sequences of the resulting PCR products were analyzed with the IMGT database (http://www.imgt.org/) to determine the variable regions of the light chain and heavy chain, which were then cloned into shuttle vectors for light chain and the Fab region of heavy chain with a C-terminal 6×His tag, respectively^56^. The resulting constructs were co-transfected to HEK-293S GnTI^−^ cells (ATCC) for expression at 37 °C. After 72 h, the medium was harvest and applied to Ni-NTA gravity columns. Following several washes with buffer A containing 20 mM imidazole, bound material was eluted with buffer A containing 250 mM imidazole. The eluate was then applied to a Superdex-200 Increase size-exclusion chromatography column (GE Healthcare) in buffer A and peak fractions containing Fab^15B2^ were collected for complex assembly.

### Cryo-EM sample preparation

To assemble the HMGCR^TM^-UBIAD1-BRIL^N102S^-Fab^BRIL^-Nb complex, purified HMGCR^TM^-UBIAD1-BRIL^N102S^ was first incubated with Amphipol A8-35 (Anatrace) for 4 h at 4 °C. The detergent was then removed by overnight incubation with Bio-beads (Bio-Rad). The amphipol-solubilized complex was mixed with Fab^BRIL^ and Nb at 1:1.5:2.25 molar ratio for 1 hour at 4 °C. The HMGCR^TM^-UBIAD1-BRIL^N102S^-Fab^BRIL^-Nb complex was finally purified with a Superose-6 column (GE Healthcare) in buffer A. Peak fractions containing the assembled complex were concentrated to ~10 mg/ml and 2 mM Fluorinated Fos-Choline-8 (Anatrace) was added to the sample before making grids. To assemble the HMGCR^TM^-UBIAD1-BRIL^N102S^-Fab^15B2^ complex, purified HMGCR^TM^-UBIAD1-BRIL^N102S^ and Fab^15B2^ were mixed at 1:1.1 molar ratio and incubated on ice for 1 h, followed by gel-filtration with a Superose-6 column (GE Healthcare) in buffer D. Peak fractions that contained the HMGCR^TM^-UBIAD1-BRIL^N102S^-Fab^15B2^ complex were concentrated to ~10 mg/ml. Preparation of HMGCR^TM^ (Δ40–55)-UBIAD1-BRIL^N102S^-Fab^15B2^ complex sample was following the same procedure.

### Cryo-EM imaging and data processing

The freshly purified complexes samples were added to Glow discharged Quantifoil R1.2/1.3 400 mesh Au holey carbon grids (Quantifoil), blotted using a Vitrobot Mark IV (FEI), and frozen in liquid ethane. The grids were imaged in a 300 kV Titan Krios (FEI) with a Gatan K3 Summit direct electron detector (Gatan). Data were collected using SerialEM^57^ at 0.83 Å/pixel or 0.842 Å/pixel. Images were recorded for 5-second exposures in 50 subframes with a total dose of ~60 electrons per Å^2^. Data were collected in super-resolution mode and the parameters of data collection are summarized in the Supplementary Table 1.

For all the three samples, Dark subtracted movie stacks were normalized by gain reference and the motion correction was performed using MotionCor2^58^. The contrast transfer function (CTF) was estimated using CTFFIND4^59^. After particle picking by crYOLO^60^, the low-quality images and false-positive particles were removed manually. For the HMGCR^TM^-UBIAD1-BRIL^N102S^-Fab^BRIL^-Nb complex, three data sets were collected. After 2D-classification of data set 1 in CryoSPARC^61^, classes with clean background were selected to generate initial models for the 3D-classification. Map from the best 3D class showing clear features of micelle, Fab and Nb were used as a model to fish out “good” particles from all the three data sets via 3D-classification. The resulting particles were subjected to the secondary 3D-classification with a mask in RELION-3^62^. Particles from the good classes were polished and 3D classified in RELION-3. The best class was selected for the final 3D-refinement in RELION-3. For HMGCR^TM^-UBIAD1-BRIL^N102S^-Fab^15B2^ complexes, good classes from the initial 2D-classification were select to generated initial models for the 3D-classification of the entire particle set in CryoSPARC. The best 3D class was 2D classified and the classes showing features of monomer or dimer were selected separately to refine the best 3D model, respectively. The resulting maps were used as models for the further 3D-classification. 3D classes containing monomeric or dimeric particles were subjected to 2D-classification in CryoSPARC to remove the heterogeneous particles. The remaining particles in the two classes were polished in RELION-3, followed by CTF refinement and final 3D refinement in CryoSPARC, respectively. For HMGCR^TM^ (Δ40–55)-UBIAD1-BRIL^N102S^-Fab^15B2^ complexes, monomeric and dimeric maps from HMGCR^TM^-UBIAD1-BRIL^N102S^-Fab^15B2^ complexes were used as models for 3D-classification in CryoSPARC. Particles from the monomeric and dimeric classes were applied to 2D-classification and a secondary 3D-classification to further exclude the bad particles. The remaining particles in the two classes were CTF refined follow by the final local 3D refinement in CryoSPARC, respectively.

### Model Construction, Refinement and Validation

For the HMGCR^TM^-UBIAD1-BRIL^N102S^-Fab^15B2^ complex, a HMGCR^TM^-UBIAD1-BRIL^N102S^ structure predicted by AlphaFold^63^ and a Fab^15B2^ structure predicted by Swiss-Model^64^ were docked into the Complex-1 map as the initial model. The refined Complex-1 structure was docked into the Complex-2a region of the Complex-2 map used as the initial model for Complex-2a. Both structure models were manually built by COOT^65^, followed by refinement in real space using PHENIX^66^ and in reciprocal space using Refmac with secondary-structure restraints and stereochemical restraints^67^. For cross-validation, the final model was refined with the half map 1 from the final 3D-refinement. The resulting model was used to calculate the model vs. map FSC curve against half map 1 and 2, respectively, using the Comprehensive validation module in PHENIX. MolProbity^68^ was used to validate the geometries of the model. Structure figures were generated using PyMOL (http://www.pymol.org), Chimera^69^ and ChimeraX^70^. The Complex-1 structure with the deletion of TMs 7-8 of UBIAD1 was docked into the Complex-2b region of the Complex-2 map as the initial model for Complex-2b. the Complex-2b structure was refined once in real space using PHENIX for figures. For the HMGCR^TM^-UBIAD1-BRIL^N102S^-Fab^BRIL^-Nb complex, HMGCR^TM^-UBIAD1-BRIL^N102S^ structure from Complex-1 and Fab^BRIL^-Nb structure from PDB: 6WW2 were docked into map as the initial model. The structure model was manually built by COOT and refined once in real space using PHENIX for figure preparation. For HMGCR^TM^ (Δ40–55)-UBIAD1-BRIL^N102S^-Fab^15B2^ complexes, structure of HMGCR^TM^-UBIAD1-BRIL^N102S^-Fab^15B2^ Complex-2a was docked into the monomeric and dimeric maps, respectively, as initial models. The structure models were manually built by COOT and refined once in real space using PHENIX for figure preparation.

### Transient Transfection and Immunoprecipitation

HEK-293S GnTI^−^ cells were maintained in suspension in FreeStyle 293 expression medium (Gibco, Cat# 12338-026) containing 2% FCS, 100 units/ml penicillin and 100 mg/ml streptomycin sulfate at 37 °C, 8% CO_2_. Cells were set up for experiments on day 0 at the density of 0.6 × 10^6^ cells per 60-mm dish. On day 1, cells were transfected with variants of pEZT-BM-FLAG-HMGCR^TM^, and pEZT-BM-StrepII-UBIAD1^N102S^ (Δ1–40, without BRIL insertion) indicated in Figure Legends using the FuGENE 6 transfection reagent (Promega, Cat# E2692), as described in ref. 8. On day 3, duplicate dishes of cells were harvested and washed with PBS. The resulting cell pellets were resuspended in buffer containing 20 mM HEPES, pH7.5, 150 mM NaCl, 0.4% sodium cholate, 0.4% DDM, 0.2% LMNG, 0.02% CHS, and protease inhibitor cocktail (Roche, Cat# 04693124001). The cell suspension was lysed by rotating for 1 hour at 4 °C followed by centrifugation at 20,000 × *g* for 15 min at 4 °C to obtain the cell lysates. Aliquots of the supernatant were added to anti-FLAG M2 affinity agarose gel (MilliporeSigma, Cat# A2220) and incubated for 2 hours at 4 °C. Aliquots of lysates and elution fractions from immunoprecipitations were subjected to SDS-PAGE and immunoblot analysis. Primary antibodies used for immunoblotting analysis included: mouse monoclonal anti-FLAG tag IgG-FLA-1 (MBL International, Cat# M185-3L, 1:3000 dilution), mouse monoclonal anti-StrepII tag IgG-5A9F9 (GenScript, Cat# A01732, 1:3000 dilution), and mouse monoclonal anti-β-Tubulin IgG-D3U1W (Cell Signaling Technology, Cat# 86298 S, 1:3000 dilution).

SV-589 (ΔUBIAD1) cells (Fig. 2e, f) were maintained as described previously^21^ and set up for experiments on day 0 at 4 × 10^5^ cells per 60-mm dish. On day 1, cells were transfected with variants of pCMV-HMGCR (TM1-8)-T7, Myc-UBIAD1 (N102S), and pEZT-BM-StrepII-UBIAD1^N102S^ (without BRIL insertion) (0.5 µg/dish) as indicated in the Figure Legends using X-tremeGENE™ HP transfection reagent (Roche) (3 µl/µg DNA). On day 3, cells were harvested, lysed, and subjected to subcellular fractionation as described^8^. HMGCR-deficient UT-2 cells (Fig. 5g, h) were maintained as described previously^71^ and set up for experiments on day 0 at 5 × 10^5^ cells per 60-mm dish. On day 1, the cells were transfected with pCMV-HMGCR-T7 (WT) or (Δ40–55) (1 µg/dish) in the absence or presence of 10–30 ng of pCMV-Insig-1-Myc using X-tremeGENETM-360 transfection reagent (Roche) (3 µl/µg DNA). The cells were depleted of sterol and nonsterol isoprenoids through incubation in medium supplemented with lipoprotein-deficient serum, 10 µM of the statin compactin, and 50 µM sodium mevalonate.

After 24 h at 37 °C, cells were harvested and subjected to subcellular fractionation as described above. Aliquots of membrane fractions isolated from transfected SV-589 (ΔUBIAD1) (Fig. 2e, f) and whole cell lysates of transfected UT-2 cells (Fig. 5g) were subjected to SDS-PAGE and immunoblot analysis was carried out with anti-T7 Tag antibody (MilliporeSigma, Cat# 69522, 1:5000 dilution), IgG-A9 a mouse monoclonal antibody against endogenous HMGCR (clone IgG-A9, MilliporeSigma, Cat# MABS1233, 1:1000 dilution), IgG-9E10 against the Myc epitope (MilliporeSigma, Cat# M4439, 1:5000 dilution), mouse monoclonal anti-StrepII Tag IgG, and anti-calnexin polyclonal antibody (Novus Biologicals, Cat# NB100-1965, 1:1000 dilution). For the immunoprecipitation experiment shown in Fig. 5h, transfected UT-2 cells were harvested, lysed in PBS containing 1% digitonin, and immunoprecipitated with anti-T7-coupled beads (MilliporeSigma, Cat# 69026), Aliquots of lysates and pellets of the immunoprecipitation were subjected to SDS-PAGE and immunoblot analysis with anti-T7 and IgG-9E10. Uncropped blots can be found in the Source Data file.

### Statistics and reproducibility

The experiments in Figs. 2d–f, 5g, h and Supplementary Figs. 2, 3a–c were repeated at least two times on different days. Similar results were obtained.

### Reporting summary

Further information on research design is available in the Nature Research Reporting Summary linked to this article.

## Supplementary information

Supplementary Information

Reporting Summary

## Data Availability

Data supporting the findings of this manuscript are available from the corresponding author upon reasonable request. The 3D cryo-EM density maps have been deposited in the Electron Microscopy Data Bank under the accession numbers EMD-27461 (complex in state 1), EMD-27460 (complex in state 2), EMDB-27475 (complex in amphipols), EMDB-27478 (Complex Δ40–55 in state 1), and EMDB-27477 (Complex Δ40–55 in state 2). Atomic coordinates for the models have been deposited in the Protein Data Bank under the accession numbers 8DJM [10.2210/pdb8DJM/pdb] (complex in state 1) and 8DJK [10.2210/pdb8DJK/pdb] (complex in state 2). Source Data underlying Figs. 2d–f and 5g, h are provided as a Source Data file. Source data are provided with this paper.

## Source data

Source Data
