# Supplementary material for: Regulated degradation of HMG CoA reductase requires conformational changes in sterol-sensing domain
Source: Nat Commun. 2022 Jul 25;13:4273. doi: 10.1038/s41467-022-32025-5 (PMC9314443; doi:10.1038/s41467-022-32025-5)
Supplement: Supplementary file 1 — Supplementary Information [file 41467_2022_32025_MOESM1_ESM.pdf]

Supplementary Information for

Regulated Degradation of HMG CoA Reductase Requires Conformational Changes in Sterol-Sensing Domain

Hongwen Chen, Xiaofeng Qi, Rebecca A. Faulkner, Marc M. Schumacher, Linda M. Donnelly, Russell A. DeBose-Boyd*, and Xiaochun Li*

*Correspondence to R.D.B. (russell.debose-boyd@utsouthwestern.edu) or Xiaochun.Li@UTSouthwestern.edu

This PDF file includes:

Supplementary Figs. 1-13
Supplementary Table 1

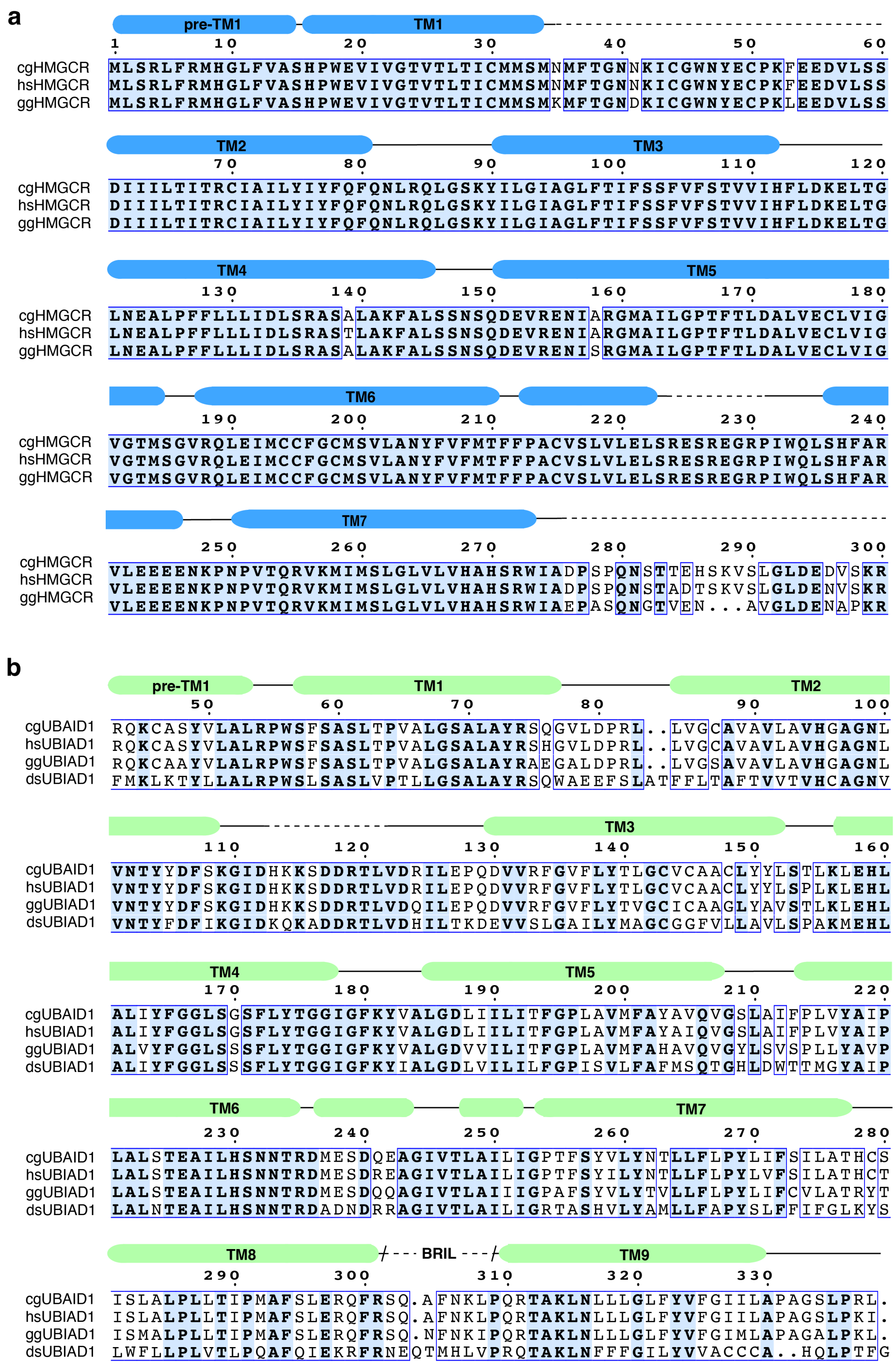

Supplementary Fig. 1 | Sequence alignments of HMGCR and UBIAD1. a, The sequence alignment of human (hs), hamster (cg) and chicken (gg) HMGCR. **b,** The sequence alignment of human (hs), hamster (cg), chicken (gg) and drosophila (ds) UBIAD1. The TMs and the residue numbers of hamster homologues are indicated above the protein sequence. The flexible regions that are not modeled in the structures are indicated by dashed lines.

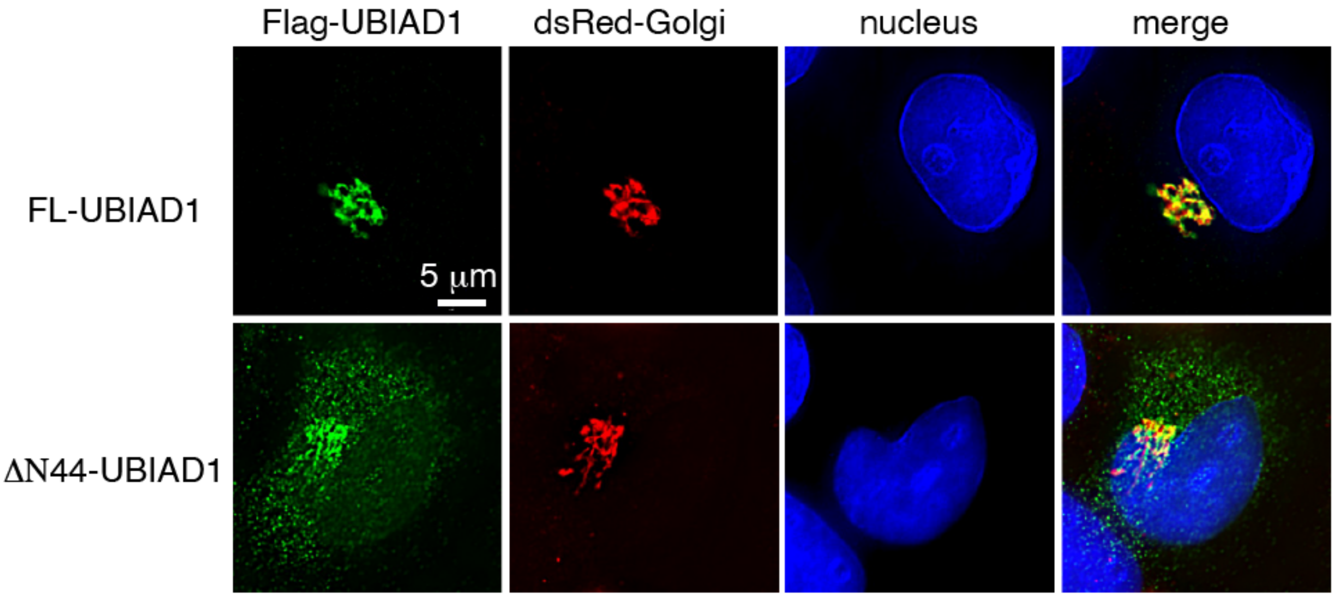

Supplementary Fig. 2 | UBIAD1 harboring 44-amino acid N-terminal deletion continues to localize to Golgi of cells. SV-589 cells transfected with expression plasmids encoding Myc-UBIAD1 (WT) or (44-338) and DsRed-Golgi in 10% fetal calf serum were fixed and analyzed by immunofluorescence deconvolution microscopy using IgG-9E10 (against transfected Myc-UBIAD1) and a 100x oil objective.

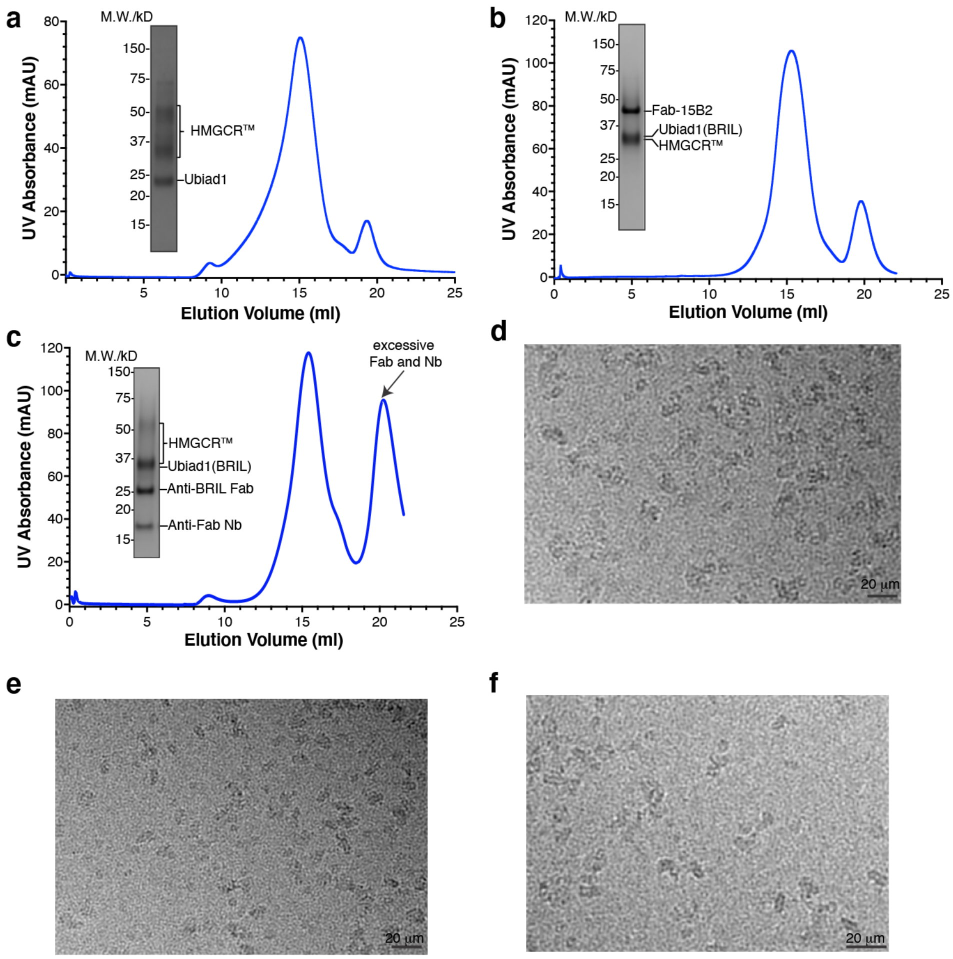

Supplementary Fig. 3 | Expression and purification of HMGCR^{TM}-UBIAD1^{N102S} complexes. Representative Superose 6 increase 10/300 gel-filtration chromatogram of HMGCR^{TM} -UBIAD1^{N102S} **(a)**, HMGCR^{TM}-UBIAD1-BRIL^{N102S}-Fab^{15B2} **(b)** and HMGCR^{TM}-UBIAD1-BRIL^{N102S}-Fab^{BRIL}-Nb^{Fab} **(c)** are shown. The peak fraction is shown on SDS-PAGE with molecular markers. Each protein is indicated**. d,** A representative electron micrograph of HMGCR^{TM}-UBIAD1-Fab^{BRIL}-Nb^{Fab}. **e,** A representative electron micrograph of HMGCR^{TM}-UBIAD1-Fab^{15B2} complex. **f,** A representative electron micrograph of HMGCR^{TM} (Δ40-55)-UBIAD1-Fab^{15B2} complex.

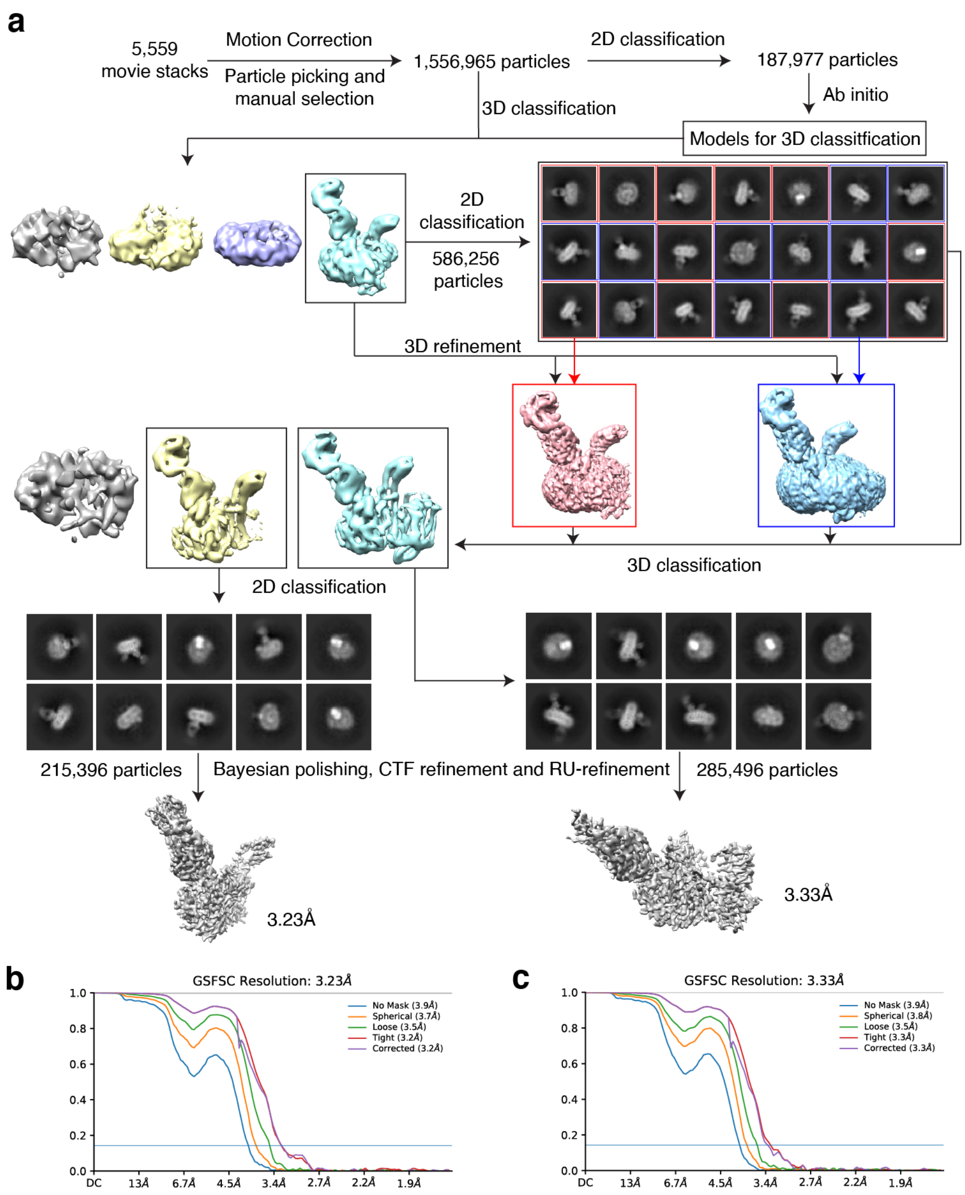

Supplementary Fig. 4 | Data processing of HMGCR^{TM}-UBIAD1-Fab^{15B2} complex. a, The data processing workflow. The cryo-EM 3D classes as well as the 2D classes are shown. The final cryo-EM map from CryoSPARC is shown. **b,** Fourier shell correlation (FSC) curve of complex 1 as a function of resolution using CryoSPARC output. **c,** Fourier shell correlation (FSC) curve of complex 2 as a function of resolution using CryoSPARC output.

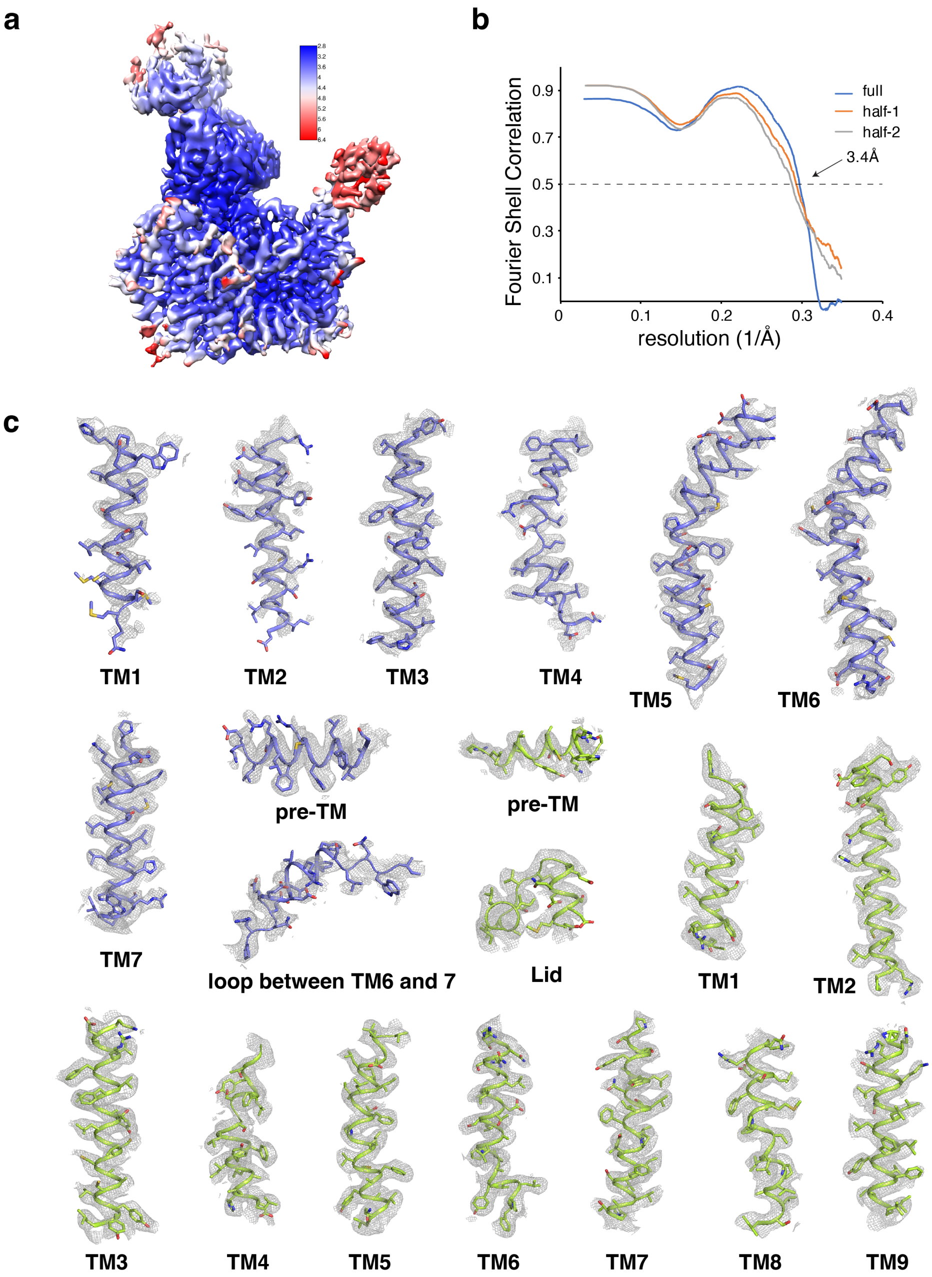

Supplementary Fig. 5 | Cryo-EM map of structural elements of HMGCR^{TM}-UBIAD1^{N102S} complex 1. a, Cryo-EM map colored by local resolution estimation using CryoSPARC. **b,** The FSC curves calculated between the refined structure model and the half map used for refinement (yellow), the other half map (gray) and the full map (blue). **c,** The major helices in HMGCR^{TM} (blue) and UBIAD1^{N102S} (green). EM density maps and models of the complex are shown in mesh and cartoon. Cryo-EM maps are shown at 5σ level.

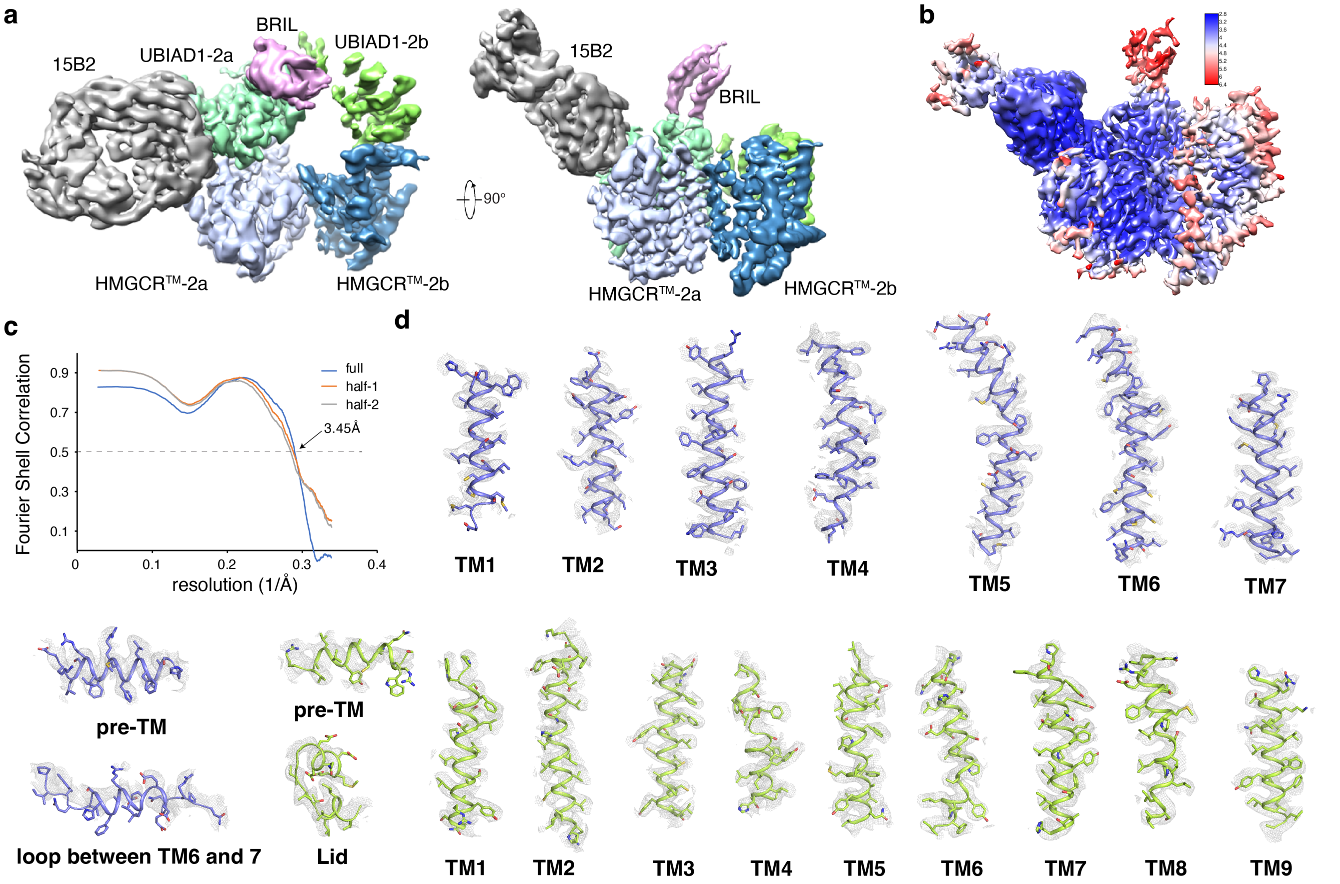

Supplementary Fig. 6 | Cryo-EM maps of structural elements of HMGCR^{TM}-UBIAD1^{N102S} complex 2a. **a,** The positions of complex 2a and complex 2b in the cryo-EM map of complex 2. **b,** Cryo-EM map colored by local resolution estimation using CryoSPARC. **c,** The FSC curves calculated between the refined structure model and the half map used for refinement (yellow), the other half map (gray) and the full map (blue). **d,** The major helices in HMGCR^{TM} (blue) and UBIAD1^{N102S} (green). EM density maps and models of the complex are shown in mesh and cartoon. Cryo-EM maps are shown at 5σ level.

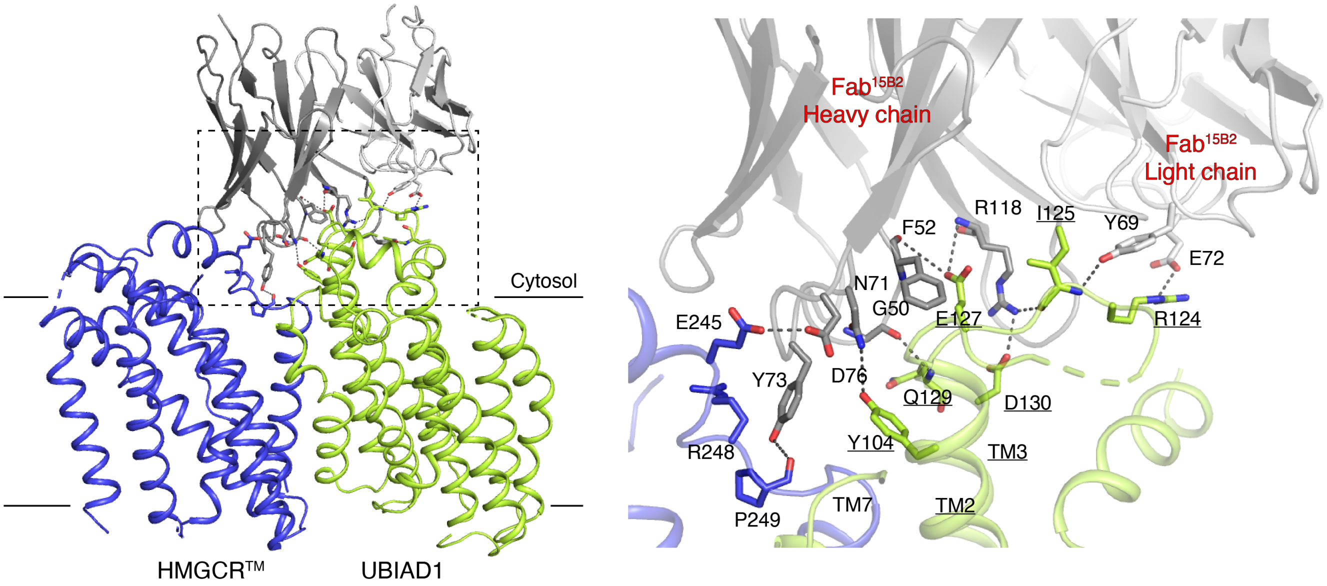

Supplementary Fig. 7 | Interaction of Fab^{15B2} with the HMGCR^{TM}-UBIAD1 complex 1. Overall structure of Fab^{15B2} bound to the HMGCR^{TM}-UBIAD1 complex 1 is shown on the left panel. Interactions between amino acids in Fab^{15B2}, UBIAD1, and HMGCR are shown on the right panel. Residues are represented as sticks; dashed lines represent hydrophilic interactions.

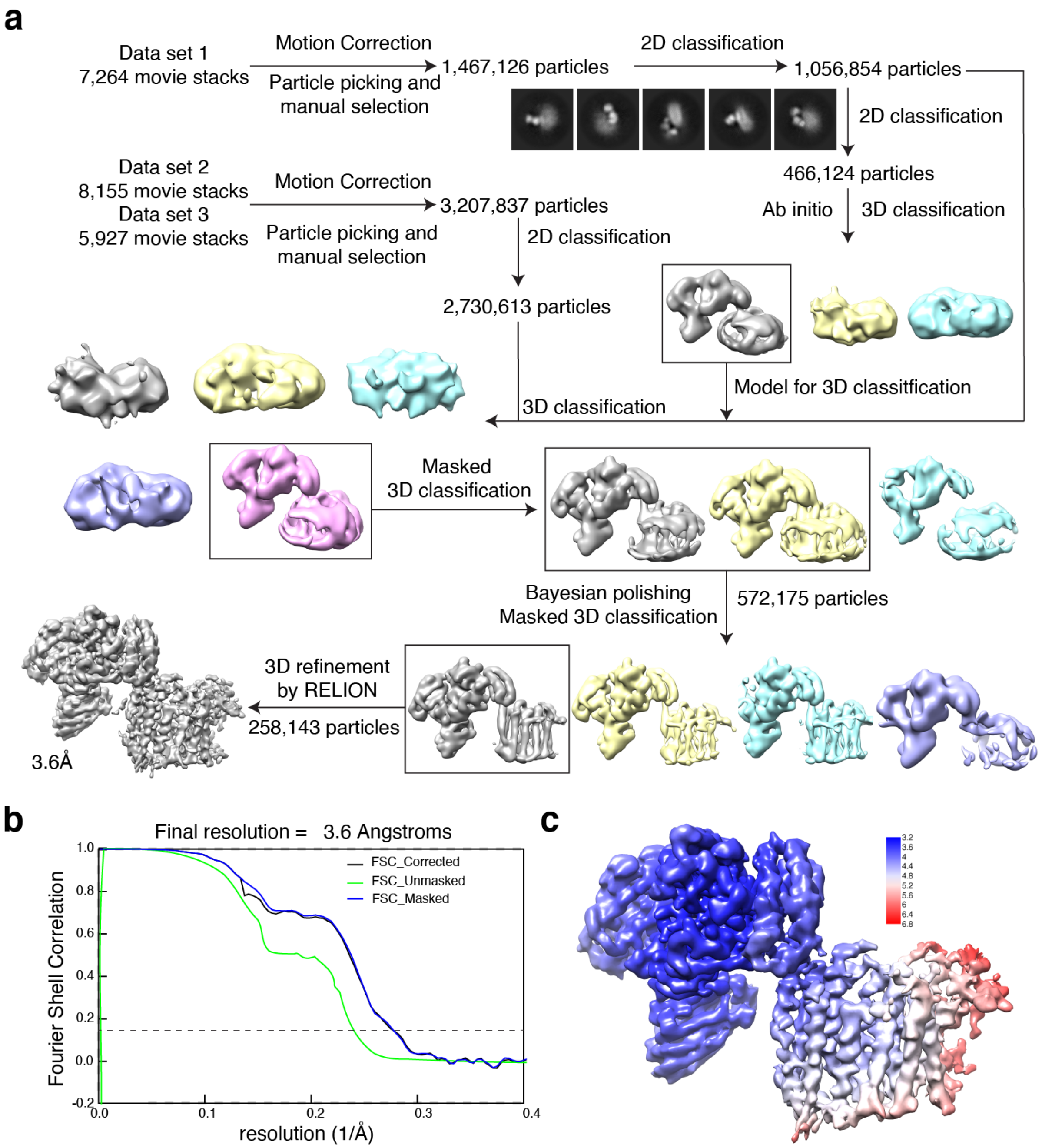

Supplementary Fig. 8 | Data processing of HMGCR^{TM}-UBIAD1-Fab^{BRIL}-Nb^{Fab} complex. a, The data processing workflow. The cryo-EM 3D classes as well as the 2D classes are shown. The final cryo-EM map from RELION is shown. **b,** Fourier shell correlation (FSC) curve as a function of resolution using RELION output. **c,** Density maps of structure colored by local resolution estimation using RELION.

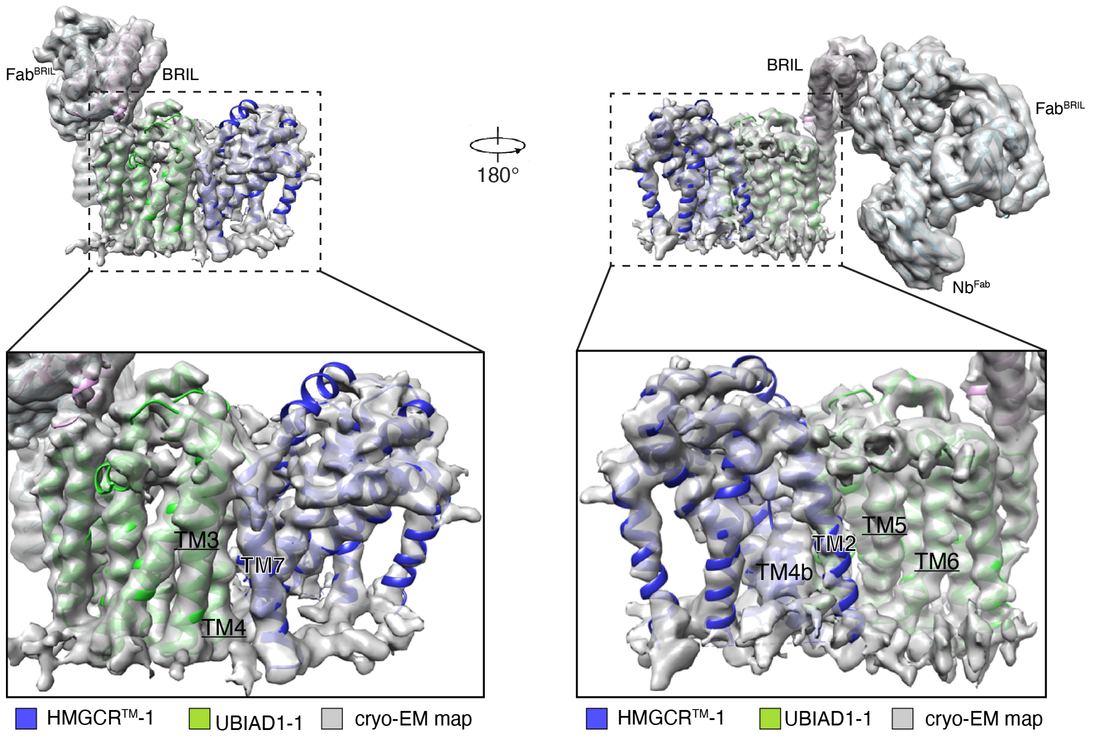

Supplementary Fig. 9 | The structure of complex 1 docked into the cryo-EM map of HMGCR^{TM}-UBIAD1-Fab^{BRIL}-Nb^{Fab} complex. The cryo-EM map is colored in gray. The Fab, Nb and BRIL are indicated.

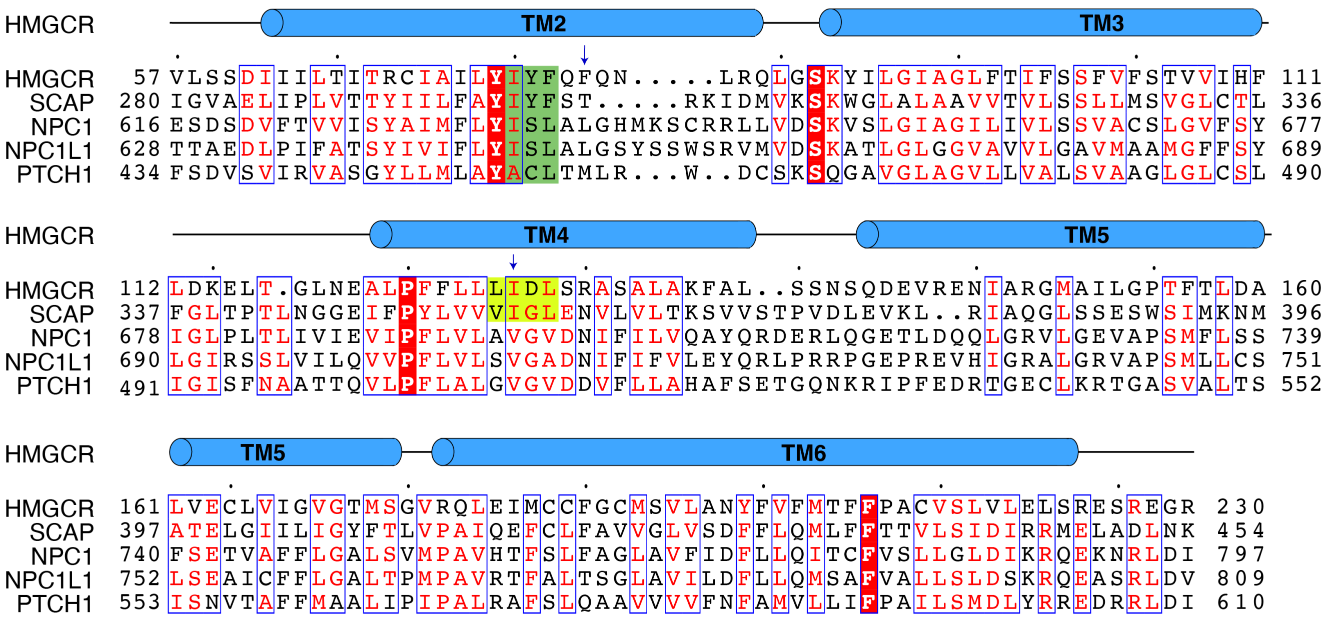

Supplementary Fig. 10 | Sequence alignment of the SSD of the SSD-containing proteins. The conserved residues are colored in red and the broken regions in TM4 of HMGCR and SCAP are highlighted in yellow. The "YIYF" motif that is required for Insig binding is colored in green. The residues F80 and I132 of HMGCR are indicated by arrows.

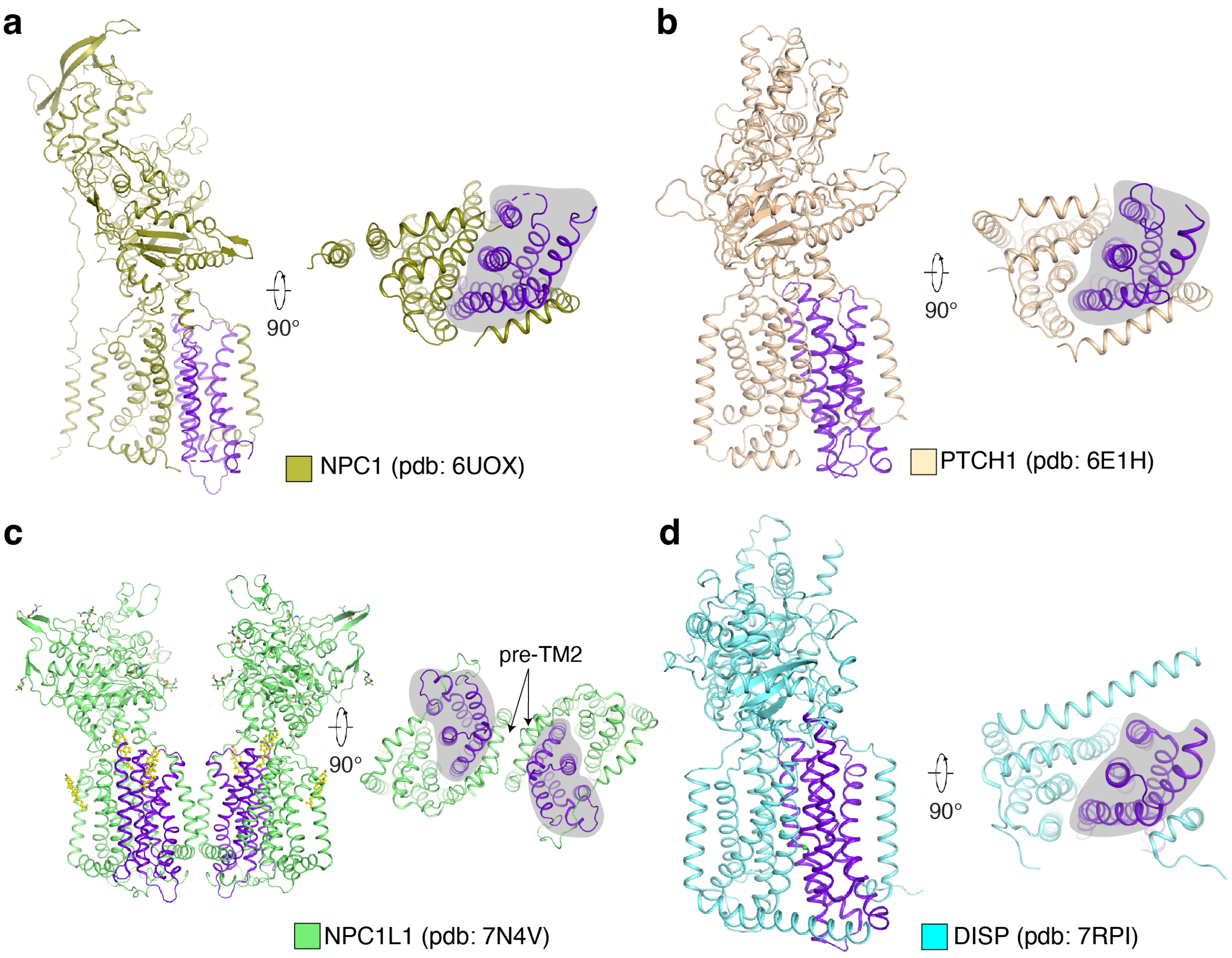

Supplementary Fig. 11 | Structures of SSD-containing proteins. **a,** Structure of NPC1. **b,** Structure of PTCH1. **c,** Structure of NPC1L1. The homodimer interface is indicated by arrows. The cholesterol molecules are shown in yellow sticks. **d,** Structure of DISP. The SSD is colored in purple and indicated by gray circles.

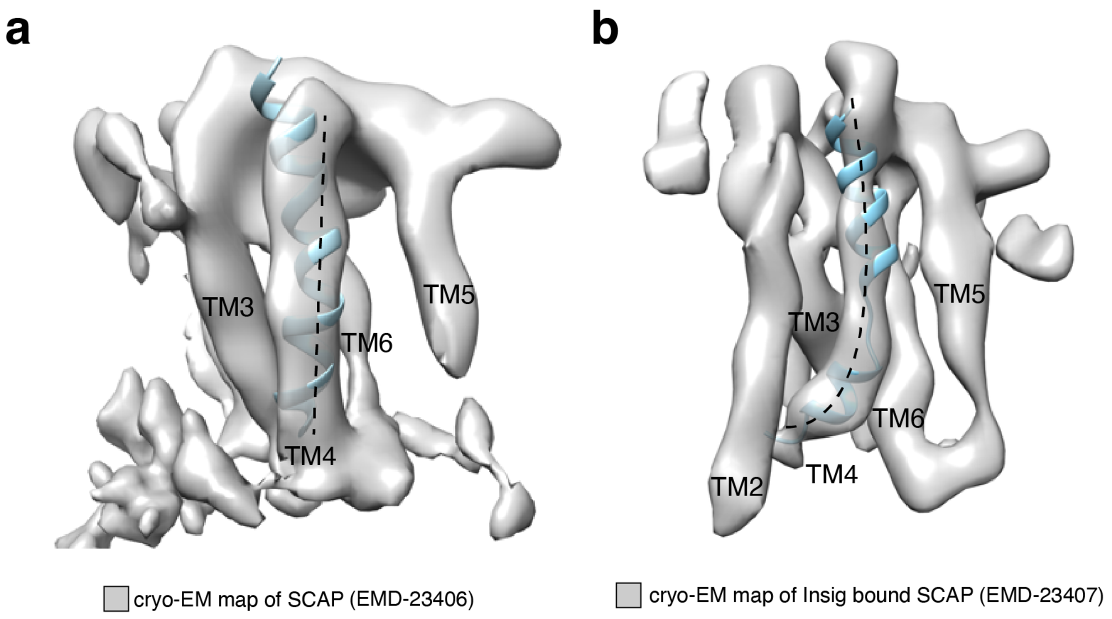

Supplementary Fig. 12 | Structures of HMGCR-TM4 modeled into cryo-EM maps of SCAP alone or when bound to Insig. **a,** The TM4 of HMGCR (Conformation B) fit into the cryo-EM map of chicken SCAP. The model reveals the TM4 straight in the membrane. TM2 of SCAP could not be determined in the map. **b,** The TM4 of HMGCR (Conformation A) fit into the cryo-EM map of Insig-bound chicken SCAP. The model reveals the TM4 bended in the membrane. TMs 2-6 are indicated.

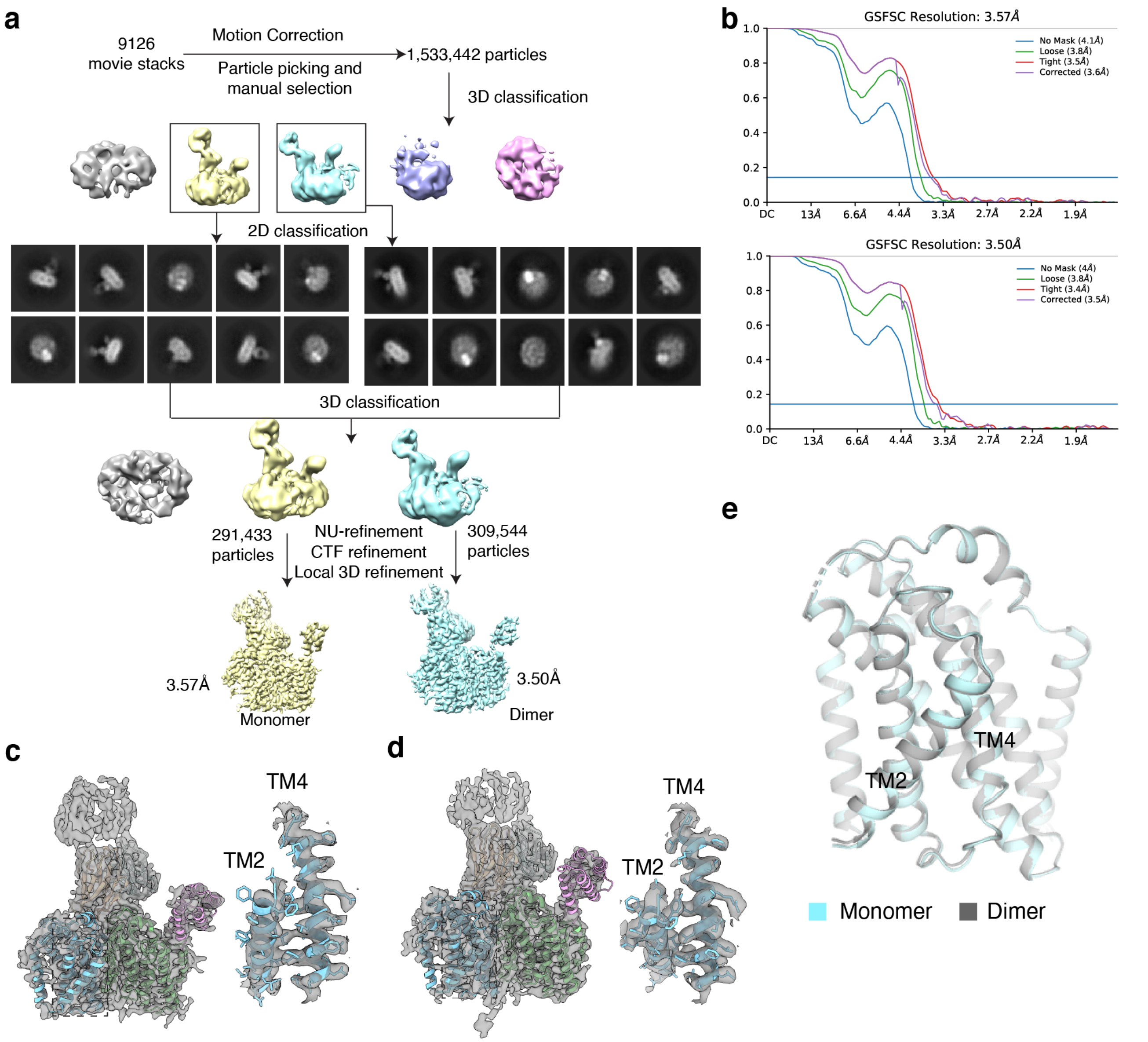

Supplementary Fig. 13 | Structures of HMGCR^{TM} (Δ40-55)-UBIAD1-Fab^{15B2} complex. **a,** The data processing workflow. The detergent micelle size in the monomeric state is much small than that of the dimeric state facilitating the identification of the two states. **b,** Fourier shell correlation (FSC) curve as a function of resolution using CryoSPARC output. **c,** Cryo-EM map of the monomeric complex. The cryo-EM maps of HMGCR-TM2 and TM4 are shown. **d,** Density maps of the dimeric complex. The cryo-EM maps of HMGCR-TM2 and TM4 are shown. **e,** Structural comparison of HMGCR^{TM} (Δ40-55) in the monomeric and dimeric states. The TM2 and TM4 share a similar conformation in both complexes.

Supplementary Table 1 | Cryo-EM data collection, refinement and validation statistics.

	Complex 1 (EMDB-27461) (PDB-8DJM)	Complex 2A (EMDB-27460) (PDB-8DJK)	Complex 2B	Complex in amphipols (EMDB-27475)	Complex Δ40-55 (EMDB-27478/27477)
**Data collection and processing**					
Magnification	59382			59382	60241
Voltage (kV)	300			300	300
Electron exposure (e–/Å^2)	61.5			61.5	61.5
Defocus range (μm)	-1.0 to -2.0			-1.0 to -2.2	-1.0 to -2.0
Pixel size (Å)	0.842			0.842	0.83
Symmetry imposed	C1	C1		C1	C1/C1
Initial particle images (no.)	1,556,965			4,674,963	1,533,442
Final particle images (no.)	215,396	285,496		258,143	291,433/309,544
Map resolution (Å) FSC threshold 0.143	3.23	3.33		3.6	3.57/3.5
**Refinement**					
Model resolution (Å) FSC threshold 0.5	3.40	3.45			
Map sharpening *B* factor (Å^2)	-117.3	-121.6			
Model composition					
Non-hydrogen atoms	7032	7091			
Protein residues	862	870			
Ligands	8	8			
B factors (Å^2)					
Protein	145.8	128.2			
Ligand	104.8	124.8			
R.m.s. deviations					
Bond lengths (Å)	0.009	0.009			
Bond angles (°)	1.396	1.406			
Validation					
MolProbity score	1.62	1.77			
Clashscore	7.04	7.61			
Poor rotamers (%)	0.69	0.82			
Ramachandran plot					
Favored (%)	96.43	94.82			
Allowed (%)	3.57	5.18			
Disallowed (%)	0.00	0.00			
