# Supplementary material for: Regulated degradation of HMG CoA reductase requires conformational changes in sterol-sensing domain
Source: Nat Commun. 2022 Jul 25;13:4273. doi: 10.1038/s41467-022-32025-5 (PMC9314443; doi:10.1038/s41467-022-32025-5)
Supplement: Supplementary file 2 — Reporting Summary [file 41467_2022_32025_MOESM2_ESM.pdf]

nature portfolio

Corresponding author(s): Xiaochun Li and Russell A. DeBose-Boyd

Last updated by author(s): Jul 2, 2022

Reporting Summary

Nature Portfolio wishes to improve the reproducibility of the work that we publish. This form provides structure for consistency and transparency in reporting. For further information on Nature Portfolio policies, see our Editorial Policies and the Editorial Policy Checklist.

Statistics

For all statistical analyses, confirm that the following items are present in the figure legend, table legend, main text, or Methods section.

n/a	Confirmed	
☒	☐	The exact sample size (*n*) for each experimental group/condition, given as a discrete number and unit of measurement
☒	☐	A statement on whether measurements were taken from distinct samples or whether the same sample was measured repeatedly
☒	☐	The statistical test(s) used AND whether they are one- or two-sided<br>*Only common tests should be described solely by name; describe more complex techniques in the Methods section.*
☒	☐	A description of all covariates tested
☒	☐	A description of any assumptions or corrections, such as tests of normality and adjustment for multiple comparisons
☒	☐	A full description of the statistical parameters including central tendency (e.g. means) or other basic estimates (e.g. regression coefficient) AND variation (e.g. standard deviation) or associated estimates of uncertainty (e.g. confidence intervals)
☒	☐	For null hypothesis testing, the test statistic (e.g. *F*, *t*, *r*) with confidence intervals, effect sizes, degrees of freedom and *P* value noted<br>*Give P values as exact values whenever suitable.*
☒	☐	For Bayesian analysis, information on the choice of priors and Markov chain Monte Carlo settings
☒	☐	For hierarchical and complex designs, identification of the appropriate level for tests and full reporting of outcomes
☒	☐	Estimates of effect sizes (e.g. Cohen's *d*, Pearson's *r*), indicating how they were calculated

Our web collection on statistics for biologists contains articles on many of the points above.

Software and code

Policy information about availability of computer code

Data collection	SerialEM 3.7.10
Data analysis	CTFFIND4 4.1.8, Relion 3.1, Refmac 5.8.0091, COOT 0.8.8, Phenix 1.13, PyMOL 1.8.6.0, MolProbity 4.3, Chimera 1.12, ChimeraX 1.3, MotionCor2 1.2.1, crYOLO 1.7.6, cryoSPARC 3.3.0

For manuscripts utilizing custom algorithms or software that are central to the research but not yet described in published literature, software must be made available to editors and reviewers. We strongly encourage code deposition in a community repository (e.g. GitHub). See the Nature Portfolio guidelines for submitting code & software for further information.

Data

Policy information about availability of data

All manuscripts must include a data availability statement. This statement should provide the following information, where applicable:

- Accession codes, unique identifiers, or web links for publicly available datasets
- A description of any restrictions on data availability
- For clinical datasets or third party data, please ensure that the statement adheres to our policy

Data supporting the findings of this manuscript are available from the corresponding author upon reasona-ble request. A reporting summary for this Article is available as a Supplementary Information file. The 3D cryo-EM density maps have been deposited in the Electron Microscopy Data Bank under the accession numbers EMD-27461 (complex in state 1), EMD-27460 (complex in state 2), EMDB-27475 (complex in amphipols), EMDB-27478 (Complex Δ40-55 in state 1), and EMDB-27477 (Complex Δ40-55 in state 2). Atomic coordinates for the atomic model have been deposited in the Protein Data Bank under the accession numbers 8DJM (complex in state 1) and 8DJK (complex in state 2).

Field-specific reporting

Please select the one below that is the best fit for your research. If you are not sure, read the appropriate sections before making your selection.

- [x] Life sciences
- [ ] Behavioural & social sciences
- [ ] Ecological, evolutionary & environmental sciences

For a reference copy of the document with all sections, see nature.com/documents/nr-reporting-summary-flat.pdf

Life sciences study design

All studies must disclose on these points even when the disclosure is negative.

Sample size	No sample size calculation was performed. The data size for cryo-EM was determined by the availability of the microscope time and the particle density on the grids. Sufficient cryo-EM data were collected to achieve the reported resolution of map, which is sufficient for model building.
Data exclusions	CryoEM data processing involved removing poor-quality or damaged particles to achieve high resolution maps through pre-established standard data classification procedures.
Replication	Each experiment was reproduced at least two times on separate occasions. Experimental findings were reliably reproduced.
Randomization	Randomization was not necessary as the independent variables to be tested were sufficient for the functional interpretations within this study. i.e. WT vs mutant vs control conditions or dose response determination.
Blinding	Blinding is not necessary or valid for the purposes of structural determination. For functional analysis, blinding was not necessary due to the quantitative nature of the experiment. All experimental data acquired in included in our statistical analysis.

Reporting for specific materials, systems and methods

We require information from authors about some types of materials, experimental systems and methods used in many studies. Here, indicate whether each material, system or method listed is relevant to your study. If you are not sure if a list item applies to your research, read the appropriate section before selecting a response.

Materials & experimental systems

n/a	Involved in the study	
[ ]	[x]	Antibodies
[ ]	[x]	Eukaryotic cell lines
[x]	[ ]	Palaeontology and archaeology
[x]	[ ]	Animals and other organisms
[x]	[ ]	Human research participants
[x]	[ ]	Clinical data
[x]	[ ]	Dual use research of concern

Methods

n/a	Involved in the study	
[x]	[ ]	ChIP-seq
[x]	[ ]	Flow cytometry
[x]	[ ]	MRI-based neuroimaging

Antibodies

Antibodies used	HMGCR-UBIAD1 Fab 15B2, anti-FLAG tag IgG-FLA-1 (MBL International, Cat#M185-3L), anti-StrepII tag IgG-5A9F9 (GenScript, Cat# A01732), anti-β-Tubulin IgG-D3U1W (Cell Signaling Technology, Cat# 86298S), anti-T7 Tag antibody (MilliporeSigma, Cat#69522), anti-calnexin polyclonal antibody (Novus Biologicals, Cat#NB100-1965), IgG-9E10 against the Myc epitope (MilliporeSigma, Cat#M4439), mouse monoclonal antibody against endogenous HMGCR (clone IgG-A9, MilliporeSigma, Cat# MABS1233).
Validation	Fab 15B2 binding to HMGCR-UBIAD1 complex was shown by the co-migration of the Fab and the transporter on the size exclusion column.<br>https://www.mblbio.com/bio/g/dtl/A/?pcd=M185-3L<br>https://www.genscript.com/antibody/A01732-THE_NWSHPQFEK_Tag_Antibody_mAb_Mouse.html<br>https://www.cellsignal.com/products/primary-antibodies/b-tubulin-d3u1w-mouse-mab/86298<br>https://www.emdmillipore.com/US/en/product/T7Tag-Monoclonal-Antibody,EMD_BIO-69522<br>https://www.novusbio.com/products/calnexin-antibody_nb100-1965<br>https://www.sigmaaldrich.com/US/en/product/sigma/m4439<br>https://www.sigmaaldrich.com/US/en/product/mm/mabs1233

Eukaryotic cell lines

Policy information about cell lines

Cell line source(s)	HEK-293S GnTI– (ATCC, CRL-3022), UT2 (UT Southwestern, Department of Molecular Genetics, RRID:CVCL_DC04), SV-589 (RRID:CVCL_RW34) and SP2-mIL6 mouse myeloma cells (ATCC, CRL-2016)
Authentication	No further authentication was performed for commercially available cell lines.
Mycoplasma contamination	periodically test negative
Commonly misidentified lines (See ICLAC register)	None of the cell lines used is listed in the database of commonly misidentified cell lines maintained by ICLAC.
